# Comparative Structural Analysis of Human DEAD-Box RNA Helicases

**DOI:** 10.1371/journal.pone.0012791

**Published:** 2010-09-30

**Authors:** Patrick Schütz, Tobias Karlberg, Susanne van den Berg, Ruairi Collins, Lari Lehtiö, Martin Högbom, Lovisa Holmberg-Schiavone, Wolfram Tempel, Hee-Won Park, Martin Hammarström, Martin Moche, Ann-Gerd Thorsell, Herwig Schüler

**Affiliations:** 1 Structural Genomics Consortium, Karolinska Institutet, Stockholm, Sweden; 2 Structural Genomics Consortium and Department of Pharmacology, University of Toronto, Toronto, Canada; University of Cambridge, United Kingdom

## Abstract

DEAD-box RNA helicases play various, often critical, roles in all processes where RNAs are involved. Members of this family of proteins are linked to human disease, including cancer and viral infections. DEAD-box proteins contain two conserved domains that both contribute to RNA and ATP binding. Despite recent advances the molecular details of how these enzymes convert chemical energy into RNA remodeling is unknown. We present crystal structures of the isolated DEAD-domains of human DDX2A/eIF4A1, DDX2B/eIF4A2, DDX5, DDX10/DBP4, DDX18/myc-regulated DEAD-box protein, DDX20, DDX47, DDX52/ROK1, and DDX53/CAGE, and of the helicase domains of DDX25 and DDX41. Together with prior knowledge this enables a family-wide comparative structural analysis. We propose a general mechanism for opening of the RNA binding site. This analysis also provides insights into the diversity of DExD/H- proteins, with implications for understanding the functions of individual family members.

## Introduction

DExD/H-box RNA helicases, from virus and bacteria to eukaryotes, play important roles in processes including ribosome biogenesis, RNA processing and folding, ribonucleoprotein (RNP) remodeling, RNA nuclear export, the regulation of RNA translation and transcription, and nonsense-mediated RNA decay. DExD/H-box RNA helicases have multiple functions in these processes: They can act as RNA chaperones, ATP-dependent RNA helicases and unwindases, as RNPases by mediating RNA-protein association and dissociation [1]–[4] or as co-activators and co-repressors of transcription ([5]–[7] and refs. therein). Cancer cell lines often feature deregulated expression or impaired functioning of RNA helicases [5], [8]. In addition, several family members are captured and regulated by viral proteins [9], are involved in viral RNA maturation [10], or mediate antiviral host defense [11], [12]. Inhibition of individual RNA helicases as a therapeutic route is currently being explored (e.g., [13]–[17]).

DExD/H-box proteins often contain accessory regulatory domains and localization modules, but their cores consist of two RecA-like domains joined by a short flexible linker. The N-terminal domain is commonly referred to as conserved domain-1, or DEAD-domain, and the C-terminal domain as conserved domain-2, or helicase domain [3], [4], [18]. Both domains contribute to the binding site for RNA substrates and both contribute to ATP hydrolysis. These activities are coupled to one another by allostery throughout the protein molecules. Consequently, a detailed understanding of how these proteins convert chemical energy into RNA remodeling requires knowledge of the structures of the two conserved domains independent of each other and interacting in the closed active state. To date, crystal structures of tandem domains are available for several DExD/H-box helicases, also in complex with RNA substrates [19]–[22]. To understand the RNA remodeling event and the underlying structural rearrangements, it is important to compare these structures with those of each domain in isolation.

We have solved crystal structures of single domains from eleven human DExD/H-box helicases of the DEAD-motif subfamily. A comparative analysis of these structures uncovered not only isoform specific features, but also nucleotide specific positioning of flexible elements that are common to several proteins. We suggest a structural mechanism for the linkage between binding of ATP and activation of the RNA binding site.

## Results and Discussion

We used X-ray crystallography to determine the structures of the DEAD-domains of DDX2A, DDX2B, DDX5, DDX10, DDX18, DDX20, DDX47, DDX52, and DDX53, as well as the helicase domains of DDX25 and DDX41. While the physiological roles of these proteins are diverse (Table 1) all structures show the RecA-like fold. Superposition of the DEAD-domain structures gives root mean square deviations of Cα-atom positions between 0.6 and 1.9 Å for proteins with sequence identity between 86 and 27%. The two helicase domains have a sequence identity of 23% and their structures superimpose with an r.m.s.d. of 3 Å. Details of the synchrotron data collection, structure determination, and refinement statistics are presented in Table 2.

**Table 1:** Summary of previously established roles and functions for the RNA helicases covered in this study.

Helicase	Function
**DDX2A**	DDX2A (eIF4A1) is essential for translation initiation. It is part of the eIF4F complex that consists of eIF4G, eIF4E and eIF4A [51]–[53]. Its activity is strongly enhanced by eIF4G, eIF4B and eIF4H [54]. The eIF4F complex and eIF4A are potential targets for anti cancer drugs [55]–[57].
**DDX2B**	Also known as eIF4A2, an isoform of DDX2A.
**DDX5**	DDX5 is a co-regulator of different transcription factors including ERα, p53, MyoD and Runx2, but ATPase/helicase activity is not required for transcriptional co-regulation. DDX5 also participates in pre-RNA processing, alternative splicing, microRNA and ribosomal RNA processing (reviewed in ref. [6]).
**DDX10**	DDX10 is probably involved in ribosome assembly. Fusion of the nucleoporin gene NUP98 with the DDX10 gene leads to the NUP98-DDX10 gene product. This fusion protein is involved in leukemogenesis [58], [59].
**DDX18**	DDX18 (Myc-regulated DEAD-box protein, or MrDP; [60]) is a nucleolar protein that is specifically upregulated in highly proliferating cells [61].
**DDX20**	DDX20 (Gemin3) is a component of the SMN (Survival of Motor Neurons) complex that is involved in assembly and reconstruction of different RNP (ribonucleoprotein) complexes [62]. DDX20, Gemin4 and eIF2C2 form a separate complex that contains numerous miRNAs [63]. DDX20 also binds to the Epstein-Barr Virus Nuclear Proteins EBNA2 and EBNA3C. The poliovirus-encoded proteinase 2A^pro^ cleaves DDX20 resulting in DDX20 inactivation and reduced snRNP assembly [64].
**DDX25**	DDX25 (GRTH) is a testis specific, gonadotropin and androgen regulated protein that is essential for completion of spermatogenesis [65]. DDX20 acts as a shuttling protein in the gene-specific nuclear export of RNA messages. Furthermore it regulates the translation of specific genes in germ cells [66].
**DDX41**	DDX41 (Abstrakt) post-transcriptionally regulates the expression levels of the insc protein that is essential for control of cell polarity and spindle orientation [67].
**DDX47**	DDX47 is involved in pre-rRNA processing. It interacts with NOP132 which recruits pre-rRNA processing proteins to the region within the nucleolus were rRNA is transcribed [68].
**DDX52**	DDX52 (Rok1) is required for the release of snR30 (small nucleolar RNA-30) from pre-ribosomes. snR30 is one of three snoRNAs that are critical for pre-rRNA processing in yeast. DDX52 ATPase activity is important for optimal pre-ribosomal RNA processing, but not essential for release of snR30 [69].
**DDX53**	DDX53 (CAGE) is expressed in testis and various tumors, but not in other tissues. Expression of the CAGE-gene is determined by its methylation status [70].

**Table 2:** Summary of crystallographic data analysis and refinement statistics*.

**Structure**	DDX2A	DDX2B	DDX5	DDX10	DDX18	DDX20	DDX25	DDX41	DDX47	DDX52	DDX53
**Domain**	DEAD	DEAD	DEAD	DEAD	DEAD	DEAD	helicase	helicase	DEAD	DEAD	DEAD
**PDB entry**	2G9N	3BOR	3FE2	2PL3	3LY5	3B7G	2RB4	2P6N	3BER	3DKP	3IUY
**Ligand**	-	-	ADP	ADP	PO_4_	AMPPNP	-	-	AMP	ADP	AMP
**Beamline**	ESRF ID14-2	APS 19-ID	BESSY 14.2	MAX II I911-2	DIAMOND I04	ESRF ID29	BESSY 14.2	ESRF ID14-4	ESRF ID29	ESRF ID23-1	ESRF ID14-2
**Wavelength (Å)**	0.93300	0.97242	0.9184	1.04123	0.9789	1.00595	0.95373	1.04005	0.97472	1.00000	0.97930
**Space group**	P 1 21 1	P 21 21 2	C 2 2 21	P 61 2 2	P 31	P 31 2 1	P 43 21 2	P 65 2 2	C 1 2 1	P 1 21 1	P 1 21 1
**Cell dimensions**											
a, b, c (Å)	a = 47.8,b = 78.25, c = 59.09	a = 58.09, b = 80.1, c = 42.74	a = 84.57, b = 106.87, c = 117.32	a = 63.5, b = 63.5, c = 304.01	a = 41.34, b = 41.34, c = 230.54	a = 63.56, b = 63.56, c = 214.6	a = 70.31, b = 70.31, c = 187.12	a = 68.01, b = 68.01, c = 305.6	a = 93.05, b = 70.37, c = 35.86	a = 40.63, b = 38.36, c = 73.84	a = 56.44, b = 61.25, c = 65.79
α, β, γ (°)	α = 90, β = 103.43, γ = 90	α = 90, β = 90, γ = 90	α = 90, β = 90, γ = 90	α = 90, β = 90, γ = 120	α = 90, β = 90, γ = 120	α = 90, β = 90, γ = 120	α = 90, β = 90, γ = 90	α = 90, β = 90, γ = 120	α = 90, β = 90.7, γ = 90	α = 90, β = 90.37, γ = 90	α = 90, β = 96.36, γ = 90
**Highest resolution shell range(Å)**	2.37–2.25	1.92–1.85	2.67–2.60	2.30–2.15	2.85–2.7	2.00–1.90	2.90–2.80	2.80–2.60	1.50–1.40	2.20–2.10	2.53–2.40
**R_meas_** (a)	0.07 (0.24)(b)	0.14 (0.67)(c)	0.14 (0.74)	0.05 (0.16)	0.24 (1.29)	0.081 (0.13)	0.11 (0.82)(b)	0.10 (0.45)	0.05 (0.14)	0.09 (0.38)	0.20 (0.61)
**I/σ(I)**	7.4 (5.3)	27.6 (3.8)(d)	11.0 (2.4)	54.1 (24.5)	6.2 (2.0)	32.10 (21.3)	15.65 (2.6)	35.9 (12.6)	28.7 (14.1)	12.8 (4.6)	13.4 (4.2)
**Completeness (%)**	99.7 (100.0)	100 (99.9)	99.4 (99.8)	99.7 (100.0)	99.98 (100.0)	99.7 (100.0)	100.0 (100.0)	99.9 (100.0)	99.5 (100.0)	99.5 (99.8)	100.0 (100.0)
**Redundancy**	7.4 (3.5)	11.4 (10.1)	4.0 (4.0)	25.4 (26.5)	5.1 (5.5)	20.5 (18.2)	8.8 (9.1)	40.0 (41.8)	7.3 (7.3)	3.7 (3.7)	9.3 (9.5)
**Refinement**											
**Resolution range (Å)**	20–2.25	30–1.85	35–2.6	30–2.15	38.4–2.8	38.43–1.90	19.44–2.80	29.72–2.60	28.24–1.40	19.56–2.10	45.13–2.40
**No. reflections**	19100	17560	15770	19842	10358	38606	11208	13220	43193	12804	17613
**R_work_/R_free_**	0.1761/0.2573	0.185/0.226	0.205/0.273	0.210/0.248	0.246/0.274	0.172/0.202	0.233/0.267	0.243/0.294	0.165/0.189	0.182/0.236	0.201/0.251
**B-factor (Å^2^)**											
Protein	15	21	27	48	47	18	82	57	13	23	23
Water	17	17	20	45	29	29	53		28	17	25
Ligand			27	43	36	26			14	12	17
**R.m.s deviations**											
Bond lengths (Å)	0.018	0.015	0.012	0.017	0.007	0.014	0.01	0.016	0.014	0.013	0.007
Bond angles (°)	1.732	1.344	1.354	1.695	0.952	1.648	1.244	1.602	1.638	1.414	1.157
**Ramachandran plot**											
Favored regions (%)(e)	96.0	99.5	98.3	98.2	96.88	98.8	91.4	95.2	99.6	99.6	98.8
Allowed regions (%)(e)	99.2	100	100	100	100	100	99.7	100	100	100	100

*Values in parentheses refer to the outermost resolution shell.

(a) R_meas_ as described in [71].

(b) R_sym_ as described in [72].

(c) Calculated using Rmerge, Version 2 [73].

(d) Calculated as the ratio of average I over average error.

(e) Determined using Molprobity [74].

**Figure 1:**
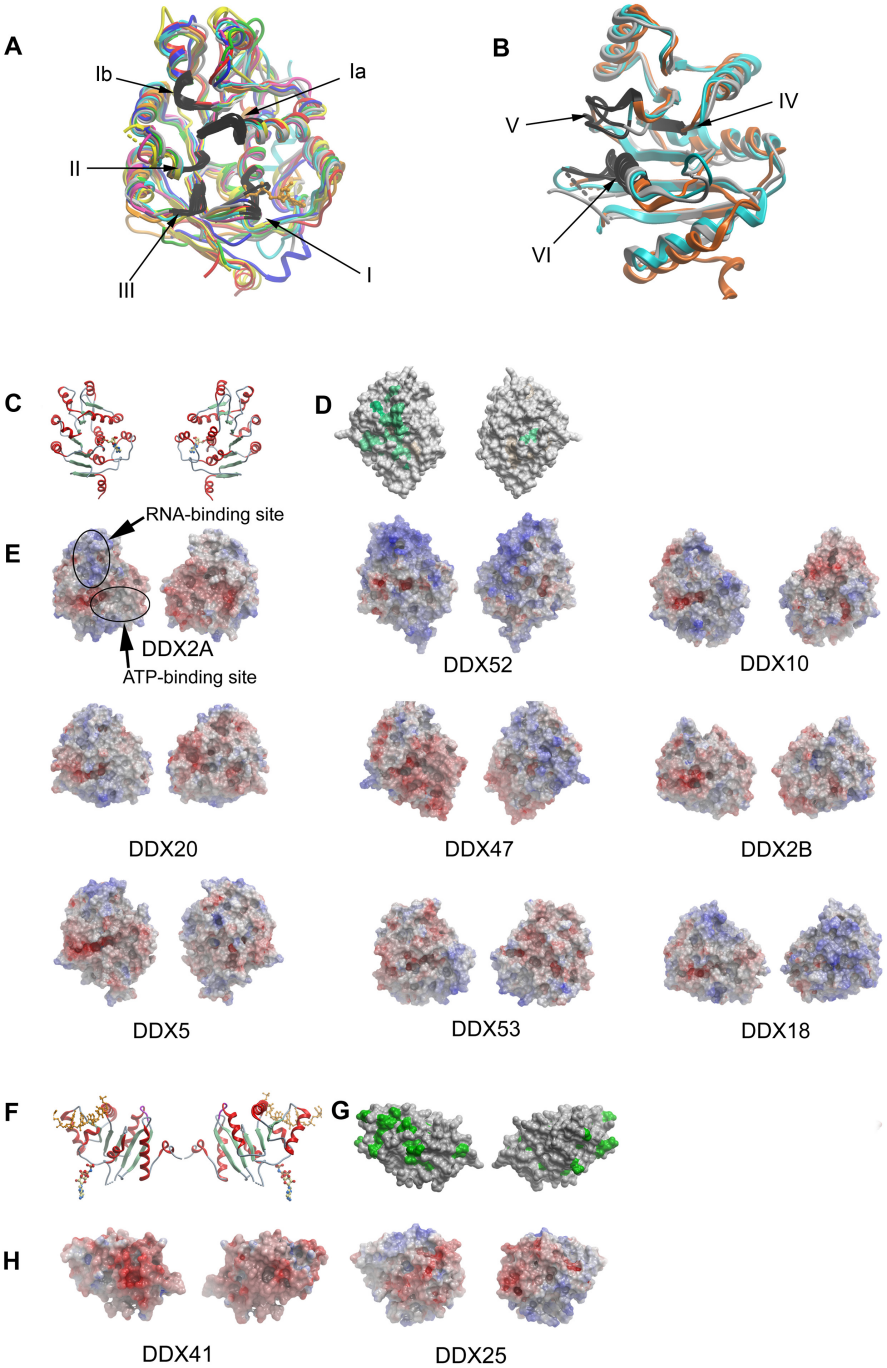
Crystal structures of DEAD-box conserved domains-1 and -2. (A) Superposition of the DEAD-domains of DDX2A (green), DDX2B (brown), DDX5 (red), DDX10 (turquoise), DDX18 (grey), DDX47 (dark blue), DDX52 (yellow), and DDX53 (dark yellow). The positions of conserved motifs I–III (black) are indicated. (B) Superposition of the helicase domains of DDX19 (light blue), DDX25 (grey) and DDX41 (orange). The positions of conserved motifs IV–VI (black) are indicated. (C) Cartoon representations of the DDX5 helicase domain in the same orientations as in the following two panels. (D) Conserved surface patches (green), projected onto the DDX47 DEAD-domain surface. (E) Electrostatic surface representation of DEAD-domains. Negative charges are shown in red and positive charges in blue. (F) Cartoon representations of the DDX41 helicase domain in the same orientation as in the following two panels. The RNA and AMPPNP (sticks representation) of the superposed DDX19 structure mark the RNA and nucleotide binding sites. (G) Conserved surface patches (green), projected onto the DDX25 helicase-domain surface. (H) Electrostatic surface representation of helicase domains.

**Figure 2:**
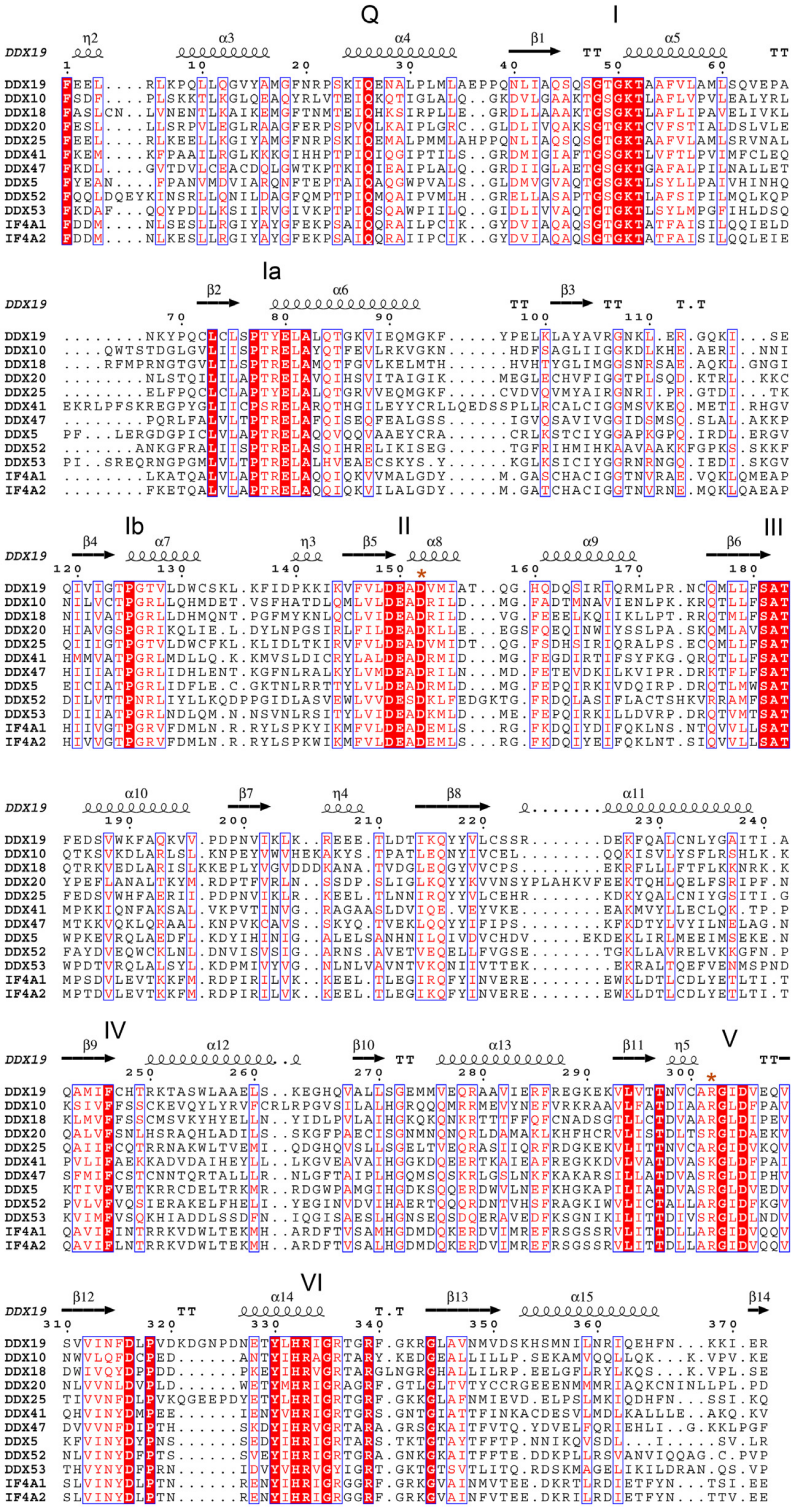
Sequence alignments of the two RecA-like domains of the DEAD-box proteins described in this study. Conserved sequence motifs are indicated. Secondary structural elements are given for DDX19 (PDB entry 3G0H) above the alignment. Asterisks mark the terminal aspartate of the DEAD motif and the arginine of motif V, the interaction of which is central to positioning of α-helix 8 (see also Figure 5C, D). Sequences shown are human DDX19B (gene accession number: 13177688); DDX10 (13514831); DDX18 (38327634); DDX20 (23270929); DDX25 (29792166); DDX41 (21071032); DDX47 (45786091); DDX5 (16359122); DDX52 (27697141); DDX53 (45709415); eIF4A1/DDX2A (16307020); and eIF4A2/DDX2B (45645183).

Superpostition of the different crystal structures illustrates the location of flexible regions (Figure 1A, 1B). In general, regions of high sequence conservation (the conserved motifs in particular) contribute to the binding sites for nucleotide and for RNA, and these sites coincide with the highest structural similarity (Figure 2). Conversely, unconserved regions in the DEAD-domains determined here show a higher r.m.s.d. in their Cα-atom positions. Some of the unconserved regions in the structures are flexible, as documented by high B-factors and partially missing electron density.

### Diverse surface properties among DEAD domains

We compared the surface charge distributions of the DEAD-domain structures (Figure 1E, 1H). All DEAD-domains feature a conserved patch that constitutes the nucleotide binding site and part of the RNA binding site. This patch forms a negatively charged channel between α-helices 8 and −10 that extends to the Mg^2+^-binding site. The negative charges originate from the side chains of the two helices, including the DEAD-motif on α-helix 8. As expected, the RNA binding cleft is positively charged in all DEAD domains, but the charged patches differ in size. The remainder of the DEAD-domain surfaces differs in electrostatic surface properties among the family members.

### ATP binding site: The flexible P-loop

**Figure 3:**
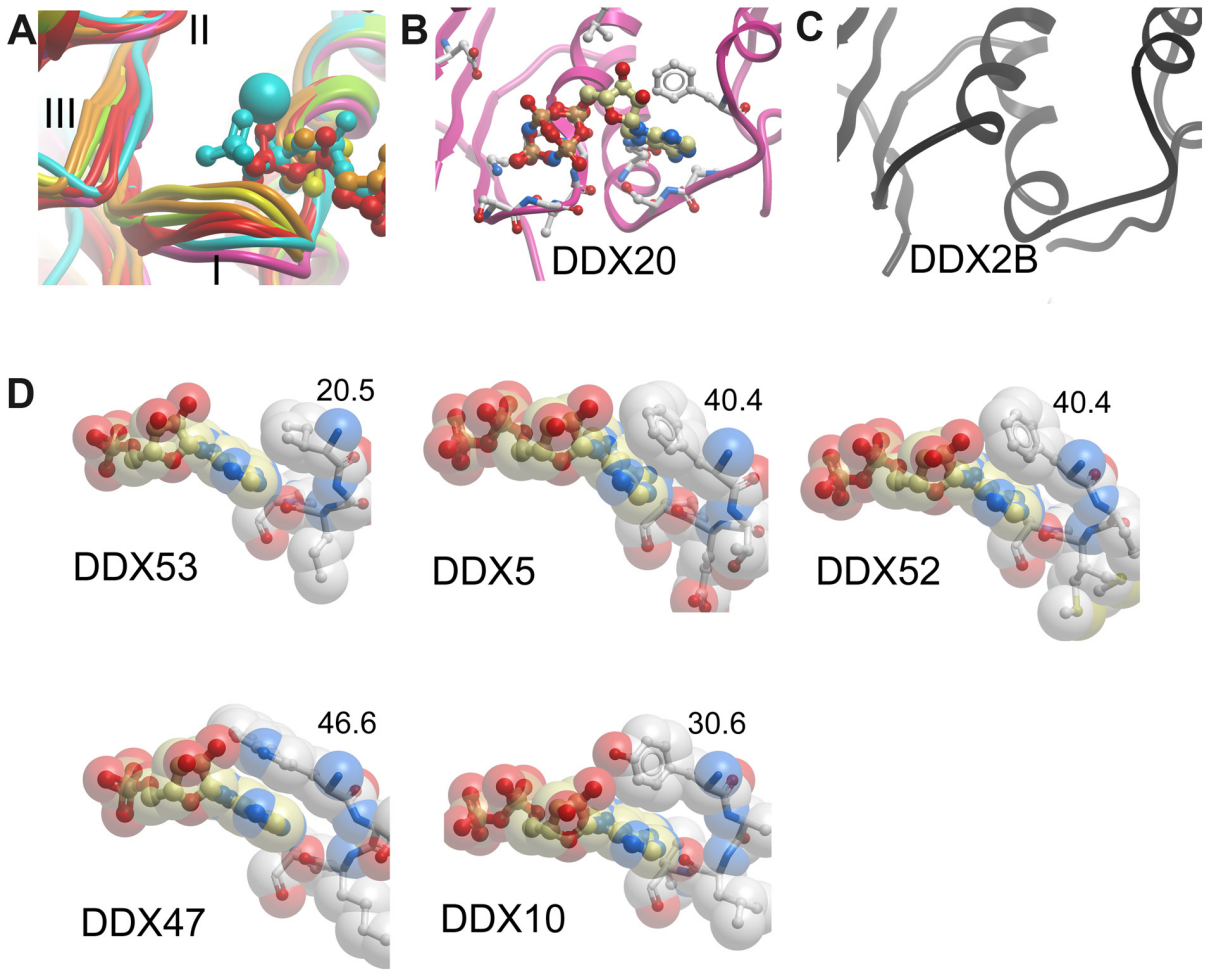
Details of the ATP binding sites. (A) Superposition of multiple DEAD-domains to illustrate variability in P-loop (Motif I) conformations. P-loops in DEAD-domain structures with bound phosphate (yellow), with bound AMP (orange), with bound ADP (red), DDX19 P-loop with bound AMPPNP and Mg^2+^ (blue), DDX20 P-loop with bound AMPPNP (magenta), and P-loop in nucleotide-free eIF4A/DDX2A (green) are shown. Motifs I, II and III are indicated. (B) Two different conformations of the β- and γ-phosphates in the DDX20-AMPPNP complex. Side chains that interact with the AMPPNP are shown as balls-and-sticks. (C) DDX2B with a closed P-loop. The α-helix that follows the P-loop starts one turn earlier compared to other DEAD-domain structures shown. (D) Variability of interactions with the adenosine nucleotide. The adenosine moiety is coordinated through π-stacking interactions or hydrophobic interactions. Numbers denote the interaction surface, in Å^2^, between the nucleotide and the stacking side chain, as determined using the PISA server [25].

Conserved motifs I (the P-loop), Ia, II, and the Q-motif participate in nucleotide binding [3], [23]. The P-loop and motif II coordinate the nucleotide phosphates and the magnesium ion, whereas residues of the Q-motif bind and recognize the adenine moiety. The side chains that participate in nucleotide and magnesium binding are highly conserved (Figure 2). The nucleotide phosphates interact with backbone atoms, a conserved lysine, and the divalent cation. Superposition of the DEAD-domains shows that the structures of the P-loop and motif III are determined by the state of nucleotide hydrolysis. The P-loop is in a wide-open conformation when ATP is bound, as seen in DDX20 as well as in the previously published structures of DDX19 [22] and eIF4AIII [19]. In the crystal complexes with either ADP or AMP the loop closes up, resulting in a shift in Cα-atom positions by up to 3.5 Å between the ATP- and the AMP-states, or by up to 2.5 Å between the ADP- and the AMP-state (Figure 3A). Thus the conformation of the P-loop is determined by the nucleotide phosphates, and longer phosphate tails result in a more open loop. This observation agrees with previous results [24]. Motif III follows the P-loop transition and its position changes by up to 3 Å toward the P-loop. Motifs Ia, Ib and II seem unaffected by the state of ATP hydrolysis, and their conformations remain unchanged even in the crystal structures in which the nucleotide binding site is not occupied.

Two of the structures show unique P-loop conformations. The DDX2B structure features an α-helix 4 that is longer than in other helicases, and leads into an unusually closed P-loop conformation (Figure 3C). As a consequence, the ATP binding site is not visible on the surface of the DDX2B structure. This conformation is most likely induced by a crystal contact in this region.

The AMPPNP-bound DDX20 structure contains no metal ion (Figure 3B). Lack of γ-phosphate coordination by a metal ion leads to a shift in the position of the β- and γ-phosphates, which bind where the α- and β-phosphates are bound in other ATP complexes. Since the adenine base is coordinated in the usual fashion the α-phosphate and the sugar moiety are tilted out of the expected positions. This illustrates that DDX20 (and presumably other helicases) can bind ATP also in the absence of divalent cation. However, a divalent cation is needed to allow coordination of three phosphates in the correct geometry for catalysis.

### Diversity in ATP coordination

Some of the side chains that interact with the nucleotides are not conserved, and most of these are found in the Q-motif. Three hydrogen bonds between the adenine ring and the protein ensure specific binding of adenosine nucleotides. These are formed by the conserved glutamine and the backbone carbonyl five residues upstream of the glutamine (Figure 3). The 6^th^ residue upstream of the conserved glutamine is an aromatic residue in most DEAD-box helicases. Its side chain stacks with the nucleotide base, stabilizing it in its position. Interestingly this residue is not conserved: While phenylalanine is most common, DDX10 has a tyrosine and DDX47 has a tryptophan in the corresponding position. Moreover, an aromatic residue in this position is not obligatory: DDX53 features an isoleucine, with weaker van-der-Waals interactions with the adenosine ring than the base stacking interactions with the aromatic side chains (Figure 3D). We analyzed the protein-nucleotide binding interfaces in these crystal structures using the PISA server [25]. This analysis showed that, while the overall ligand interface areas are similar in the different nucleotide complexes, the contribution by the base stacking residues vary considerably. The variability in the stacking residue position may reflect different needs for conformational flexibility in this region of the DEAD-domains.

### Helicase domain variation

The helicase domain contributes to nucleotide coordination *via* motifs V and VI. From the closed state DDX19 structure [22] it is apparent that four side chains are of particular importance: The aspartate of motif V coordinates the O3′ of the ribose. The second arginine side chain of motif VI (HRxGRxGR) interacts with the γ-phosphate. The third arginine, which is also the putative arginine finger during ATP hydrolysis, coordinates all three ATP phosphates. The variable residue that follows this arginine coordinates the adenosine ring by different means. In the DDX19 helicase domain a phenylalanine stacks with the adenosine rings. A superposition of DDX19 with the DDX25 and DDX41 helicase domains shows that in the latter two structures part of motif VI is not visible in the electron density, indicating its flexibility. The conserved motifs IV and VI superpose well, whereas motif V shows different conformations in all three structures (Figure 1B).

The only part of motif IV that is not flexible is the histidine-arginine pair, and it superposes in all three crystal structures. The arginine points to a negatively charged pocked formed in part by side chains from motifs IV and V in the inside of the helicase domain. The aliphatic part of the arginine side chain makes a hydrophobic contact with the phenylalanine of motif IV. In the two-domain closed state structures the histidine interacts with the SAT motif from the helicase domain. Therefore, the SAT motif is indirectly linked to the ATP binding site as well as to the RNA binding sites of both domains. This explains the central importance of this motif in the coupling of ATP hydrolysis and RNA unwinding [26]. In SAT-motif mutants of eIF4A the ATPase and helicase activities were uncoupled [27]: SAT-to-AAA mutant protein is capable of binding RNA in an ATP dependent manner, but lacks RNA unwinding activity.

### Conserved and variable parts constitute the RNA binding site

**Figure 4:**
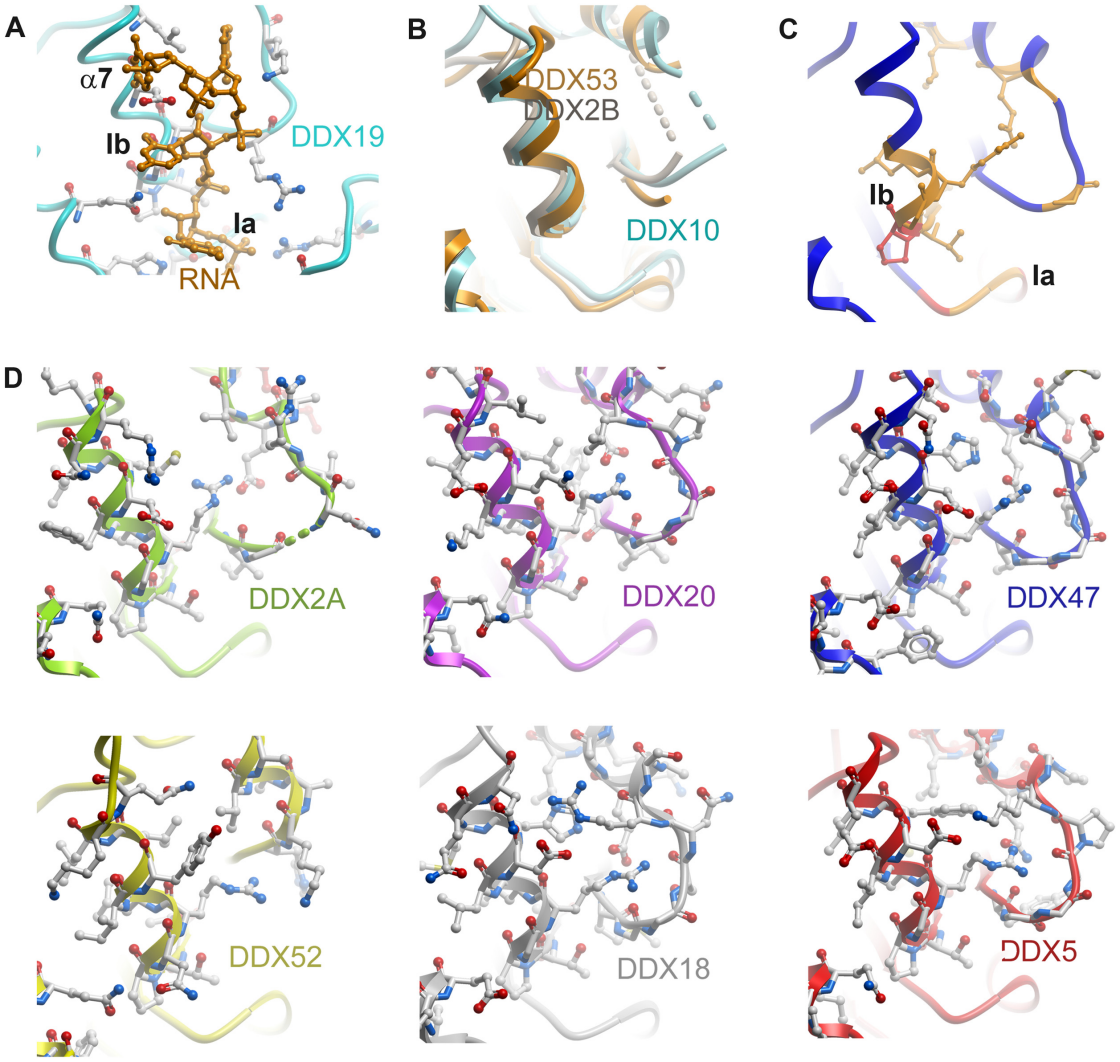
RNA binding cleft on DEAD domains. (A) DDX19 (light blue; PDB entry 3G0H) with bound RNA (light orange). RNA-interacting side chains are shown. (B) Flexible regions in DDX2B, DDX10 and DDX53 for which the electron density was not visible. (C) Sequence conservation in the RNA binding cleft, mapped onto the DDX47 structure (red, conserved; orange, partly conserved). (D) RNA binding sites of selected DEAD-domains to illustrate their sequence variation.

The available atomic resolution structures of DEAD-box helicases with bound RNA [19]–[22] show that the DEAD-domain contributes to RNA binding through two conserved and one variable structural element: (i) Motif Ia; (ii) α-helix 7, with its conserved motif Ib; and (iii) the variable loop connecting β-sheets 3 and 4. These interactions are illustrated for DDX19 in Figure 4A: While the variable loop clamps the RNA substrate in a specific conformation, motifs Ia and Ib each coordinate an RNA-backbone phosphate and induce a tilt of one or more RNA bases.

Conserved motifs Ia and Ib of DDX19 and all DEAD-domain structures described here superimpose perfectly (Figure 4). This leads us to conclude that RNA substrates are bound in a similar conformation by the conserved motifs of all these DEAD-domains. The variability in part of the RNA binding sites (Figure 4D), on the other hand, implies that different helicases could stabilize specific RNA conformations. In addition, variable side chain contribution may also reflect optimal recognition of specific nucleotide sequences.

Inspection of the RNA complexes of DDX19, vasa, and eIF4AIII [19]–[22] shows that the conserved motif that makes the most extensive contacts with the RNA-backbone phosphates is motif Ib. In two of our DEAD-domain crystal structures, anions from the crystallization buffers are bound to motif Ib (a sulfate in DDX5, and a phosphate in DDX47) highlighting the ability of this motif to bind polyanions.

### Mechanism for unblocking of the RNA binding site

**Figure 5:**
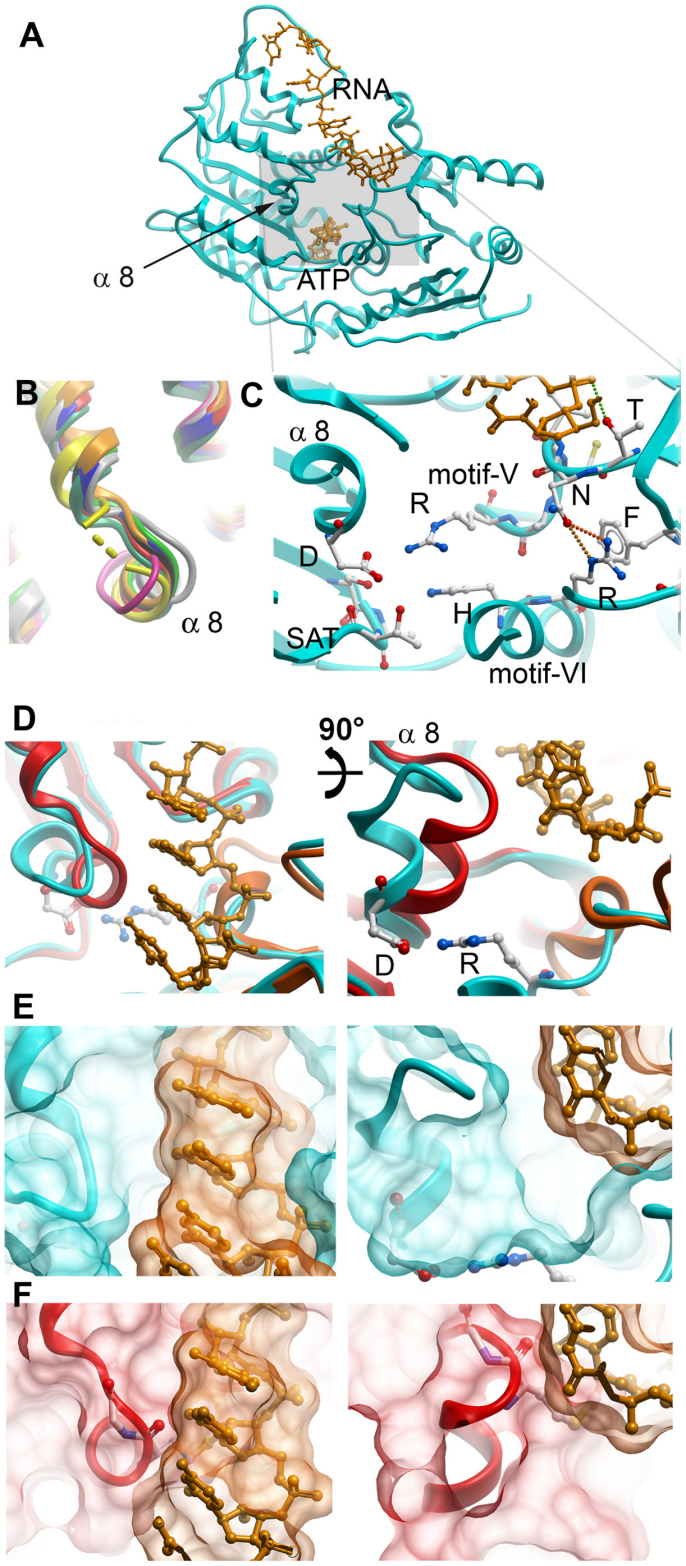
Details of the RNA binding cleft. (A) DDX19 closed state structure (PDB entry 3G0H). DDX19-bound RNA, Mg^2+^-ion and AMPPNP are in orange. (B) Superposition of several DEAD domain structures showing a conserved conformation of α-helix 8. (C) Interactions between the DEAD and helicase domains of DDX19. (D) “Top-down” view of the open and closed RNA binding cleft. DDX5 (red), the ATP-state of DDX19 (blue) and DDX41 (orange) are shown. RNA (superposed from the DDX19 complex structure) is shown in light orange. (E) Surface representation of the DDX19-RNA complex. Note that α-helix 8 does not come in contact with the RNA substrate. (F) Surface representation of DDX5 and the superposed RNA from the DDX19 complex structure. Note that α-helix 8 would clash with the RNA substrate.

Our crystal structures of both DEAD-domains and helicase domains in isolation reveal that the RNA binding site on each domain is in a conformation that is incompetent to bind RNA substrate. In the free helicase domain structures motif V, an important RNA backbone interaction site [19]–[22] is in a binding incompetent conformation. In the closed state, an RNA binding competent conformation is stabilized by the interaction of the conserved arginine of motif V with the C-terminal aspartic acid of the DEAD-motif (Figure 5C). In all single DEAD-domain structures, α-helix 8 has adopted a position that would block the RNA binding site. By contrast, upon cleft closure in the two-domain ATP analog and RNA complexes, α-helix 8 has moved out of the RNA binding site (Figure 5).

Thus, superposition of single DEAD-domain structures onto the closed state structures of DDX19 and eIF4AIII suggests involvement of α-helix 8 in the formation of a competent RNA binding site. How is α-helix 8 displaced to allow access to the RNA substrate binding site? No direct interaction between α-helix 8 and the RNA have been observed; thus displacement of α-helix 8 by the RNA substrate itself seems unlikely. Also, binding of ATP itself cannot cause α-helix 8 rotation out of the RNA site: The DEAD-motif is the only link between the nucleotide and α-helix 8, but the state of nucleotide hydrolysis does not influence the conformation of the DEAD motif (motif II; Figure 1A, 3A).

**Figure 6:**
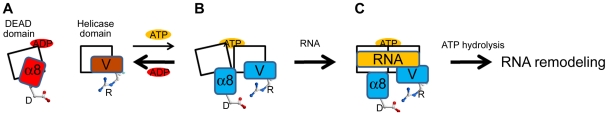
Schematic model for the regulation of RNA binding by α-helix 8 of DEAD-box helicases. (A) In the isolated domains, reflecting the open and substrate free states, the RNA binding sites are partially blocked by α-helix 8 in the DEAD-domain and motif V in the helicase domain. The aspartate indicated in the DEAD-domain is the second D of the DEAD sequence in motif II. The arginine indicated in the helicase domain is a conserved residue in motif V. Both residues are marked by asterisks in Fig 2. (B) Binding of ATP favors closure of the cleft, facilitating interaction of α-helix 8 with motif V across the cleft, thereby removing the blockage of the RNA binding site. (C) The closed cleft conformation is stabilized by RNA substrate to the competent site, allowing ATP hydrolysis to proceed.

Instead, we propose direct involvement of the helicase domain in the activation of the RNA binding site on the DEAD-domain: In the complex structures, the conserved arginine of motif V in the helicase domain forms a salt bridge with the C-terminal aspartic acid of the DEAD-motif, which is also the terminal residue of α-helix 8 (Figure 5C, 5D). This interaction stabilizes a conformation where α-helix 8 is rotated out of the RNA binding site (Figure 5D). We propose that ATP binding primes the helicases for RNA substrate binding by bringing the domains together to allow motif V to push α-helix 8 out of the RNA site on the DEAD-domain. RNA binding to the DEAD-domain then completes cleft closure to allow formation of an active ATPase site (Figure 6).

This model of cleft closure and helicase activation through regulation of α-helix 8 can reconcile published data. Moreover, it can explain how substrate release in the post-hydrolysis state is achieved. DEAD-box helicases typically bind ADP with higher affinity that ATP [28]–[31], and binding of ATP and RNA are cooperative [31]–[35]. Thus, the binding energy of the RNA-protein interaction likely stabilizes a strained conformation that is competent for ATP hydrolysis. Conversely, relief of this strain upon ATP hydrolysis and phosphate release likely drives RNA substrate remodeling [36]. According to our comparative structural analysis, ATP hydrolysis and phosphate release would allow α-helix 8 to move back into its original position, releasing the RNA substrate and switching back to a binding incompetent RNA site on the DEAD domain.

DExH-box RNA helicases differ in some aspects from the DEAD-motif containing helicases. The hepatitis C virus DExH-box helicase NS3 binds RNA in the absence of ATP [37]. DExH helicase NPH-II unwinds RNA in a processive fashion [38] and thus stays bound to the RNA after each unwinding step. Our model for the role of α-helix 8 in cleft closure of DEAD-proteins is consistent also with these properties of DExH-box RNA helicases. Whereas α-helix 8 is conserved in all DEAD-box proteins, it is missing in the DExH-box proteins (refs. [37], [39]–[42] and references therein). Moreover, the DEAD-motif aspartic acid side chain that mediates opening of the RNA binding site (Figure 5) is replaced by the histidine of the DExH-motif. Thus apparently, in the absence of α-helix 8 that may block the RNA site, this terminal aspartic acid is redundant, and the histidine that substitutes it fulfills a different function [42]. We conclude that DEAD- and DExH-box helicases differ significantly in the coupling of the RNA binding event to the conformational cycle of the two RecA domains.

## Materials and Methods

All proteins were expressed in *Escherichia coli* as N-terminally hexahistidine tagged fusion proteins, and purified by nickel affinity chromatography and gel filtration. Proteins were crystallized in sitting drops at 4°C or 20°C. X-ray diffraction data were collected at the APS (Chicago, USA), the BESSY (Berlin, Germany), the Diamond (Oxfordshire, UK), the ESRF (Grenoble, France), and the MaxLab (Lund, Sweden) synchrotron radiation facilities. Data were indexed and integrated using XDS [43], MOSFLM [44], or DENZO [45], and scaled using XSCALE [43], SCALA [46] or SCALEPACK [45]. Structures were solved by molecular replacement using PHASER [47] or MOLREP [48], and refined using REFMAC [49]. Refinement rounds were complemented with manual rebuilding using COOT [50].

Detailed Materials and Methods can be found in Table S1.

### Accession codes

The coordinates have been deposited in the Proteins Data Bank with accession codes 2G9N, 3BOR, 3FE2, 2PL3, 3LY5, 3B7G, 2RB4, 2P6N, 3BER, 3DKP, and 3IUY.

## Supporting Information

Table S1 Materials and methods detailing protein expression and purification, crystallization, X-ray data processing.
(0.15 MB PDF)
