# Supplementary material for: Comparative Structural Analysis of Human DEAD-Box RNA Helicases
Source: PLoS One. 2010 Sep 30;5(9):e12791. doi: 10.1371/journal.pone.0012791 (PMC2948006; doi:10.1371/journal.pone.0012791)
Supplement: Table S1 — Materials and methods detailing protein expression and purification, crystallization, X-ray data processing. (0.15 MB PDF) [file pone.0012791.s001.pdf]

**Structure**	DDX2A/eIF4A
**PDB Entry**	2G9N
**Entry clone accession**	gi|16307020
**Entry clone source**	Mammalian Gene Collection (BC009585)
**Tag**	N-terminal hexahistidine tag with integrated TEV protease cleavage site: mhhhhhhssgvdlgtenlyfq*sm
**Construct boundaries**	E20 – K238
**Construct sequence**	mhhhhhhssgvdlgtenlyfq*smEGVIESNWNEIVDSFDDMNLSESLLRGIYAYGFEKPSAIQQRAILPCIKGYDVIAQA QSGTGKTATFAISILQQIELDLKATQALVLAPTRELAQQIQKVVMALGDYMGASCHACIGGTNVRAEVQKLQ MEAPHIIVGTPGRVFDMLNRRYLSPKYIKMFVLDEADEMLSRGFKDQIYDIFQKLNSNTQVVLLSATMPSDVL EVTKKFMRDPIRILVKK
**Vector**	pNIC-Bsa4
**Expression host**	*E.coli* BL21(DE3)
**Growth method**	20 mL TB was inoculated with cells and 100 µg kanamycin/mL and grown overnight at 30°C. The inoculation culture was added to 1.5 L TB (supplemented with 50 µg kanamycin/mL) in 2 L bottles. The flasks were incubated in the LEX system-water bath at 37°C until the OD_{600} reached 1. At this time the flask was transferred to an 18°C water bath in the LEX-system. The Protein expression was induced by addition of 0.5 mM IPTG. The cells were incubated for approximately 18 hours.
**Extraction buffers**	50 mM Na Phosphate pH 7.5, 10 % glycerol, 0.5 mM TCEP and 500 mM NaCl, 10mM imidazole
**Extraction procedure**	The Cells were harvested by centrifugation in a SLC-6000 rotor for 10 minutes at 5000 rpm (OD 600 8.2; WCV 24.7 g). The Pellets were suspended in 90 mL Extraction buffer supplemented with a tablet of Complete EDTA-free protease inhibitor (Roche Biosciences). The Suspended cells were stored at -80°C until further use. 50 µL of ADP (200 mM) and 4 µL of benzonase (250U/µL, Novagen) was added to the suspended cells. The cells were lysed by passing them 2 times through a high pressure homogenizer (Stansted, at 5000 Psi) The sample was spun for 30 min at 20500 rpm in a Sorvall SA-800 rotor. The soluble fraction was decanted and filtered through a 0.45 µm syringe filter.
**Purification buffers**	IMAC Bind/Wash Buffer: 50 mM Na Phosphate, 10 % glycerol, 0.5 mM TCEP, 500 mM NaCl, 10mM imidazole pH 7.5. IMAC-wash and elution buffers were the same except for that the imidazole concentration was raised to 25 and 500 mM respectively. Gel filtration buffer: 20 mM HEPES, 300 mM NaCl, 10% glycerol, 0.5 mM TCEP, pH 7.5.
**Columns**	IMAC: Nickel-charged 1 mL HiTrap Chelating HP (GE Healthcare)<br>Gel filtration: HiLoad 16/60 Superdex 75 Prep Grade (GE Healthcare)
**Purification procedure**	Protein purification was performed as a two-step process on an ÄKTAxpress system (GE Healthcare) at 4°C. The IMAC column was charged with filtered lysate and washed with IMAC wash1 buffer followed by IMAC wash2 buffer. Bound protein was eluted with IMAC elution buffer and loaded onto the gel filtration column. Target protein containing fractions were pooled and TCEP was added to a final concentration of 2 mM. The protein was concentrated using an Amicon Ultra-15 centrifugal filter device with 10,000 NMWL (Millipore) to 16.2 mg/mL in a volume of 1.2 mL, and quick-frozen in liquid nitrogen.
**Post-purification**	**Tag removal**: The TEV-cleavage was performed by adding 400 µl His-tagged TEV (30 µM) to 10 mg protein in a total volume of 0.6 mL Gel filtration buffer with 2 mM TCEP for approximately 18 hours at 4°C. The Cleaved protein was loaded onto a His-trap column, detected in the flow-through and concentrated in AmiconUltra MWCO 10000 concentrators to a concentration of 19.2 mg/mL.
**Reductive methylation**	Reductive methylation of 5 mg TEV-cleaved protein was performed according to the manufacturer´s instructions (JBS Methylation Kit, JenaBioscience) in a final volume of 1 mL Gel filtration buffer containing 2 mM TCEP. The methylated protein was concentrated to 18.1 mg/mL before setting up drops.
**Crystallization**	The protein was crystallized by the sitting drop vapor diffusion method: 200 nL of the methylated protein was mixed with 100 nL of the reservoir solution consisting of 200 mM ammonium nitrate and 20 % (w/v) PEG3350. Crystal clusters grew within three days. For data collection a single crystal was separated from the cluster and soaked in a cryoprotectant solution consisting of 20 mM HEPES pH 7.5, 500 mM NaCl, 200 mM ammonium nitrate, 20 % (w/v) PEG3350 and 20 % Glycerol before flash cooling in liquid nitrogen.
**Data processing**	Intensity integration was performed by MOSFLM. Reflections were scaled with SCALA The structure was solved by molecular replacement using MOLREP with the pdb-entry 1QDE as search model. The Structure was refined with REFMAC5.

**Structure**	DDX2B/eIF4AII
**PDB Entry**	3BOR
**Entry clone accession**	gil45645183
**Entry clone source**	NP_001958.2
**Tag**	mhhhhhhssgrenlyfq*g
**Construct boundaries**	G22 – E240
**Construct sequence**	mhhhhhhssgrenlyfqgGVIESNWNEIVDNFDDMNLKESLLRGIYAYGFEKPSAIQQRAIIPCIKGYDVIAQAQSGTGK<br>TATFAISILQQLEIEFKETQALVLAPTRELAQQIQKVILALGDYMGATCHACIGGTNVRNEMQKLQAEAPHIVV<br>GTPGRVFDMLNRRYLSPKWIKMFVLDEADEMLSRGFKDQIYEIFQKLNTSIQVVLLSATMPTDVLEVTKKFMR<br>DPIRILVKKE
**Vector**	pET28-mhl
**Expression host**	*E.coli* BL21-CodonPlus(DE3)-RIL
**Growth method**	Terrific Broth
**Extraction buffers**	20 mM HEPES, pH 8.0, 300 mM NaCl, 5mM Imidazole, 0.5% CHAPS, 1 mM TCEP
**Extraction procedure**	Frozen cells were thawed and suspended in 150 mL of binding buffer supplemented with protease inhibitor cocktail (Sigma-Aldrich) and benzonase (Sigma-Aldrich), and lysed using a Microfluidizer (French press).
**Purification buffers**	Binding buffer: 20 mM HEPES pH 8.0, 300 mM NaCl, 5 mM imidazole, 1 mM TCEP.<br>Washing buffer: 20 mM HEPES pH 8.0, 150 mM NaCl, 50 mM imidazole, 1 mM TCEP.<br>Elution buffer: 20 mM HEPES pH 8.0, 150 mM NaCl, 500 mM imidazole, 1 mM TCEP<br>Gel filtration buffer: 20 mM HEPES pH 8.0, 150 mM NaCl, 1 mM TCEP
**Purification procedure**	The lysate was centrifuged at 16,000 rpm for 60 minutes and the supernatant was passed through two open columns filled with DE52 and 5 mL 50% Ni-NTA beads at 4 °C. The beads were washed using washing buffer and the proteins eluted using 16 mL elution buffer. The eluate was loaded onto a Superdex-75 gel filtration column. Eluted fractions were pooled and concentrated using amicon centrifugal filter (MW cutoff 10,000). The purity of the protein was higher than 95% judged by SDS-PAGE.
**Crystallization**	Protein stock concentration: 13.6 mg/mL, in 20mM HEPES pH 8.0, 150mM NaCl, 5% glycerol, 1 mM TCEP.<br>Crystallization: The protein was mixed with chymotrypsin (1.0 mg/mL) at 1:100 ratio (w/w) and hanging drop vapor diffusion experiments were set up. Crystallization condition: 25.45% PEG3350, 7.3 pH, 0.2 M ammonium acetate
**Data processing**	Intensity integration was performed by DENZO. Reflections were scaled with SCALEPACK. The structure was solved by molecular replacement using Phaser. The Structure was built and refined with ARP/wARP, REFMAC5 and coot.

**Structure**	DDX5
**PDB Entry**	3FE2
**Entry clone accession**	gil16359122
**Entry clone source**	Mammalian Gene Collection (BC016027)
**Tag**	N-terminal hexahistidine tag with integrated TEV protease cleavage site: mhhhhhhssgvdlgtenlyfq*sm
**Construct boundaries**	R69 – L307
**Construct sequence**	mhhhhhhssgvdlgtenlyfq*smRTAQEVETYRRSKEITVRGHNCPKPVLNFYEANFPANVMDVIARQNFTEPTAIQAQGWPVALSGLDMVGVAQTGSGKTLSYLLPAIVHINHQPFLERGDGPICLVLAPTRELAQQVQQVAAEYCRACRLKSTCIYGGAPKGPQIRDLERGVEICIATPGRLIDFLECGKTNLRRTTYLVLDEADRMLDMGFEPQIRKIVDQIRPDRQTLMWSATWPKEVRQLAEDFLKDYIHINIGALEL
**Vector**	pNIC-Bsa4
**Expression host**	*E.coli* BL21(DE3) R3 pRARE, where R3 denotes a derivative of BL21(DE3) resistant to a strain of T1 bacteriophage (SGC Oxford) and the pRARE plasmid originating from the Rosetta strain (Novagen) supplies tRNAs for rare codons.
**Growth method**	Cells from a glycerol stock were grown in 20 mL TB supplemented with 8 g/l glycerol, 100 µg/mL kanamycin and 34 µg/mL chloramphenicol at 30 ºC overnight. 20 mL of the overnight culture were used to inoculate 1.5 L TB supplemented with 8 g/l glycerol, 50 µg/mL kanamycin and approximately 500 µl Dow Corning anti-foam RD emulsion 63213 4D (BDH Silicone Products). The culture was grown in a LEX bioreactor system (Harbinger Biotechnology) at 37 ºC until the OD_{600} reached ~1.2. The bottle was down-tempered to 18 ºC over a period of 1 hour before target expression was induced by addition of 0.5 mM IPTG. Expression was allowed to continue overnight and cells were harvested the following morning by centrifugation (4,400 x *g*, 10 min, 4 ºC). The resulting cell pellet (29.6 g wet cell weight) was resuspended in lysis buffer (1.5 mL/g cell pellet), supplemented with 2000 U Benzonase (Merck) and one tablet of Complete EDTA-free protease inhibitor (Roche Applied Science). The cell suspension was stored at -80 ºC.
**Extraction buffers**	Lysis buffer: 100 mM HEPES, 500 mM NaCl, 10% glycerol, 10 mM imidazole, 0.5 mM TCEP, pH 8.0
**Extraction procedure**	The cell suspension was thawed and diluted to 2 x 68 mL with lysis buffer. Cells were disrupted by sonication (Vibra-Cell, Sonics) at 80% amplitude for 3 min effective time (pulsed 4s on, 4s off) and cell debris was removed by centrifugation (49,000 x *g*, 20 min, 4 ºC). The supernatant was decanted and filtered through a 0.45 µm flask filter.
**Purification buffers**	IMAC wash1 buffer: 20 mM HEPES, 500 mM NaCl, 10% glycerol, 10 mM imidazole, 0.5 mM TCEP, pH 7.5<br>IMAC wash2 buffer: 20 mM HEPES, 500 mM NaCl, 10% glycerol, 25 mM imidazole, 0.5 mM TCEP, pH 7.5<br>IMAC elution buffer: 20 mM HEPES, 500 mM NaCl, 10% glycerol, 500 mM imidazole, 0.5 mM TCEP, pH 7.5<br>Gel filtration (GF) buffer: 20 mM HEPES, 500 mM NaCl, 10% glycerol, 0.5 mM TCEP, pH 7.5
**Columns**	IMAC: Nickel-charged 1 mL HiTrap Chelating HP (GE Healthcare)<br>Gel filtration: HiLoad 16/60 Superdex 75 Prep Grade (GE Healthcare)
**Purification procedure**	Protein purification was performed as a two-step process on an ÄKTAxpress system (GE Healthcare) at 4°C. The IMAC column was charged with filtered lysate and washed with IMAC wash1 buffer followed by IMAC wash2 buffer. Bound protein was eluted with IMAC elution buffer and loaded onto the gel filtration column. Target protein containing fractions were pooled and TCEP was added to a final concentration of 2 mM. The protein was concentrated using an Amicon Ultra-15 centrifugal filter device with 10,000 NMWL (Millipore) to 17.6 mg/mL in a volume of 1.2 mL, and quick-frozen in liquid nitrogen.
**Post-purification**	**Tag removal**: The N-terminal histidine tag was proteolytically removed by incubating the protein with His-tagged TEV at a molar ratio of 30:1 at 4 ºC overnight. The proteolytic reaction went near to completion, as judged by SDS-PAGE. Target protein was purified from tag and protease by passing the reaction mixture over an IMAC column pre-equilibrated with IMAC wash1 buffer. The cleaved protein was concentrated and the buffer was changed to GF buffer containing 2 mM TCEP using a Vivaspin 20 centrifugal filter device with 10,000 MWCO (Sartorius). The final protein concentration was 17.5 mg/mL in a volume of 0.7 mL. The mass of the protein was confirmed by mass spectrometry.
**Crystallization**	Crystals were obtained by the sitting drop vapor diffusion method. 0.2 µl of the protein sample (diluted to 15.8 mg/mL) including 20 mM ADP and 10 mM $MgCl_2$ was mixed with 0.1 µl of well solution consisting of 0.1 M Bis-Tris pH 6.4, 0.25 M lithium sulfate monohydrate, 17.5% PEG 3350. The plate was incubated at 4 ºC and crystals appeared within 10 hours. The Cryoprotectant solution containing 0.1 M Bis-Tris pH 5.5, 0.2 M lithium sulfate, 25% PEG 3350, 0.2 M NaCl and 15% glycerol was added directly to the drop. The Crystals were mounted and flash-frozen in liquid nitrogen.
**Data processing**	Intensity integration was performed by XDS. Reflections were scaled with XSCALE. The structure was solved by molecular replacement using MOLREP with human DDX3X protein structure (2I4I) residues 3-241 as a search model. The structure was refined with REFMAC5.

**Structure**	DDX10
**PDB Entry**	2PL3
**Entry clone accession**	gil13514831
**Entry clone source**	Mammalian Gene Collection (BC093656)
**Tag**	N-terminal hexahistidine tag with integrated TEV protease cleavage site: mhhhhhhssgvdlgtenlyfq*sm
**Construct boundaries**	Q47 – A280
**Construct sequence**	mhhhhhhssgvdlgtenlyfq*smQVERESISRLMQNYEKINVNEITRFSDFPLSKKTLKGLQEAQYRLVTEIQKQTIGLA LQGKDVLGAAKTGSGKTLAFLVPVLEALYRLQWTSTDGLGVLIISPTRELAYQTFEVLRKVGKNHDFSAGLII GGKDLKHEAERINNINILVCTPGRLLQHMDETVSFHATDLQMLVLDEADRILDMGFADTMNAVIENLPKKRQ TLLFSATQTKSVKDLARLSLKNPEYVWVHEKA
**Vector**	pNIC-Bsa4
**Expression host**	*E.coli* BL21-Gold(DE3)pRARE2, where BL21-Gold(DE3) cells (Stratagene) have been transformed with pRARE2 originating from the Rosetta2 strain (Novagen). The pRARE2 plasmid supplies tRNAs for rare codons.
**Growth method**	Cells from a glycerol stock were streaked onto a LB-agar plate. 5-10 colonies were used to inoculate 25 mL TB supplemented with 8 g/L glycerol, 100 µg/mL kanamycin and 34 µg/mL chloramphenicol at 30 ºC overnight. 20ml of the overnight culture was used to inoculate 1.5 l TB supplemented with 8 g/L glycerol, 50 µg/mL kanamycin and approximately 200 BREOX FMT 30 anti-foam solution (Cognis Performance Chemicals UK Ltd). The culture was grown in a LEX bioreactor system (Harbinger Biotechnology) at 37 ºC until the OD_{600} reached ~1.2. The culture was down-tempered to 18 ºC over a period of 1 hour before target expression was induced by addition of 0.5 mM IPTG. The Expression was allowed to continue overnight and cells were harvested the following morning by centrifugation (5,500 x *g*, 10 min, 4 ºC). The resulting cell pellet (19.8 g wet cell weight) was resuspended in lysis buffer (1 mL/g cell pellet), supplemented with 0.5 tablet of Complete EDTA-free protease inhibitor (Roche Applied Science). The cell suspension was stored at -80 ºC.
**Extraction buffers**	Lysis buffer: 50 mM HEPES, 500 mM NaCl, 10% glycerol, 10 mM imidazole, 0.5 mM TCEP, pH 7.8.
**Extraction procedure**	The cell suspension was thawed and 1000 U Benzonase (Merck) was added. Cells were disrupted by sonication (Vibra-Cell, Sonics) at 80% amplitude for 3 min effective time (pulsed 4s on, 4s off) and cell debris was removed by centrifugation (49,000 x *g*, 20 min, 4 ºC). The supernatant was decanted and filtered through a 0.45 µm flask filter.
**Purification buffers**	IMAC wash1 buffer: 30 mM HEPES, 500 mM NaCl, 10% glycerol, 10 mM imidazole, 0.5 mM TCEP, pH 7.5<br>IMAC wash2 buffer: 30 mM HEPES, 500 mM NaCl, 10% glycerol, 25 mM imidazole, 0.5 mM TCEP, pH 7.5<br>IMAC elution buffer: 30 mM HEPES, 500 mM NaCl, 10% glycerol, 500 mM imidazole, 0.5 mM TCEP, pH 7.5<br>Gel filtration (GF) buffer: 30 mM HEPES, 500 mM NaCl, 10% glycerol, 0.5 mM TCEP, pH 7.5
**Columns**	IMAC: Nickel-charged 1 mL HiTrap Chelating HP (GE Healthcare)<br>Gel filtration: HiLoad 16/60 Superdex 75 Prep Grade (GE Healthcare)
**Purification procedure**	Protein purification was performed as a two-step process on an ÄKTAxpress system (GE Healthcare) at 4°C. The IMAC column was charged with filtered lysate and washed with IMAC wash1 buffer followed by IMAC wash2 buffer. Bound protein was eluted with IMAC elution buffer and loaded onto the gel filtration column. Target protein containing fractions were pooled and TCEP was added to a final concentration of 2 mM. The protein was concentrated using an Amicon Ultra-15 centrifugal filter device with 10,000 NMWL (Millipore) to 7.3 mg/mL in a volume of 1.2 mL, and snap-frozen in liquid nitrogen.
**Post-purification**	**Tag removal**: The N-terminal histidine tag was proteolytically removed by incubating the protein with His-tagged TEV protease in a molar ratio of 30:1 at 4 ºC for one hour. The proteolytic reaction went to completion, as judged by SDS-PAGE. The target protein was diluted 1:1 in GF-buffer. The protein was purified from the tag and the protease by adding Ni-NTA resins to the reaction mixture. The protein was concentrated and the buffer was exchanged to GF buffer (with 2 mM TCEP) using a centrifugal filter device with 10,000 MWCO. The final protein concentration was 18.7 mg/mL in a volume of 0.33 mL. The mass of the protein was confirmed by mass spectrometry.
**Crystallization**	Crystals were obtained by the sitting drop vapor diffusion method. 20 mM ADP and 10 mM $MgCl_2$ was added to the protein sample and 0.1 µl of the protein solution (diluted to 16.6 mg/mL) was mixed with 0.1 µl of well solution consisting of 0.1 M Tris pH 8.5, 50 mM sodium chloride and 20% ethanol. The plate was incubated at 4 ºC. The crystals appeared within one day and continued to grow for one more week to reach their maximal size (approx. 160 µm × 50 µm × 50 µm). The crystal was quickly transferred to a cryoprotectant solution consisting of well solution and 40% glycerol (replaced the same volume of water) and flash frozen in liquid nitrogen.
**Data processing**	Intensity integration was performed by XDS. Reflections were scaled with XSCALE. The structure was solved by molecular replacement using the Hera N-terminal domain from *Thermus thermophilus* as a search model (PDB entry: 2GXS) with the program MOLREP. Refmac5 was used for refinement and Coot for model building. TLS restrained refinement using 4 TLS groups was used in the refinement process. The TLS groups were selected using the tlsmd server http://skuld.bmsc.washington.edu/~tlsmd/.

**Structure**	DDX18
**PDB Entry**	3LY5
**Entry clone accession**	gi|38327634
**Entry clone source**	Mammalian Gene Collection (BC024739)
**Tag**	N-terminal hexahistidine tag with integrated TEV protease cleavage site: mhhhhhhssgvdlgtenlyfq*sm
**Construct boundaries**	N149 - G387
**Construct sequence**	mhhhhhhssgvdlgtenlyfq*smNNVEKPDNDEDESEVPSLPLGLTGAFEDTSFASLCNLVNENTLKAIKEMGFTNMTE IQHKSIRPLLEGRDLLAAAKTGSGKTLAFLIPAVELIVKLRFMPRNGTGVLILSPTRELAMQTFGVLKELMTHH VHTYGLIMGGSNRSAEAQKLGNGINIIVATPGRLLDHMQNTPGFMYKNLQCLVIDEADRILDVGFEEELKQIIK LLPTRRQTMLFSATQTRKVEDLARISLKKEPLYVG
**Vector**	pNIC-Bsa4
**Expression host**	*E.coli* BL21-Gold(DE3)pRARE2, where BL21-Gold(DE3) cells (Stratagene) have been transformed with pRARE2 originating from the Rosetta2 strain (Novagen). The pRARE2 plasmid supplies tRNAs for rare codons.
**Growth method**	The Cells from a glycerol stock were grown in 50 mL TB supplemented with 8 g/L glycerol, 100 µg/mL kanamycin and 34 µg/mL chloramphenicol at 37 ºC overnight. The overnight culture was used to inoculate 3 l TB (divided into 4 x 0.75 l bottles) supplemented with 8 g/L glycerol, 50 µg/mL kanamycin. The culture was grown in a LEX bioreactor system (Harbinger Biotechnology) at 37 ºC until the OD_{600} reached ~1.2. The culture was down-tempered to 18 ºC over a period of 1 hour before target expression was induced by addition of 0.5 mM IPTG. The Expression was allowed to continue overnight and the cells were harvested the following morning by centrifugation (4,400 x *g*, 10 min, 4 ºC). The resulting cell pellet (65 g wet cell weight) was then stored at -80 ºC.
**Extraction buffers**	50 mM HEPES, 500 mM NaCl, 10% glycerol, 10 mM imidazole, pH 7.8
**Extraction procedure**	The resulting cell pellet was resuspended in lysis buffer (1.5 mL/g cell pellet), supplemented with Protease-Inhibitors[1] and Benzonase[1] at 4°C. Cells were disrupted by sonication (Vibra-Cell, Sonics) at 80% amplitude for 3 min effective time (pulsed 4s on, 4s off) and cell debris was removed by centrifugation (49,000 x *g*, 40 min, 4 ºC). The supernatant was filtered through a 0.45 µm flask filter.<br>[1] complete stock solution: 1 tablet Complete EDTA-free (protease inhibitor) and 8 µL Benzonase (2000 U) dissolved in 1 mL buffer. 1 mL complete stock solution is added to cells from 1.5 L cell culture.
**Purification buffers**	IMAC lysis buffer: 50 mM Hepes, 500 mM NaCl, 10% glycerol, pH 7.5, 0.5 mM TCEP, 10 mM imidazole<br>IMAC wash 1 buffer: 30 mM Hepes, 500 mM NaCl, 10% glycerol, pH 7.5, 0.5 mM TCEP, 10 mM imidazole<br>IMAC wash 2 buffer: 30 mM Hepes, 500 mM NaCl, 10% glycerol, pH 7.5, 0.5 mM TCEP, 25 mM imidazole<br>IMAC elution buffer: 30 mM Hepes, 500 mM NaCl, 10% glycerol, pH 7.5, 0.5 mM TCEP, 500 mM imidazole<br>Gel filtration buffer: 30 mM Hepes, 500 mM NaCl, 10% glycerol, pH 7.5, 2 mM TCEP
**Columns**	IMAC: Nickel-charged 5 mL HisTrap HP (GE Healthcare)<br>Gel filtration: HiLoad 16/60 Superdex 75 Prep Grade (GE Healthcare)
**Purification procedure**	Protein purification was performed as a two-step process on an ÄKTAxpress system (GE Healthcare) at 4°C. The IMAC column was charged with filtered lysate and washed with IMAC wash1 buffer followed by IMAC wash2 buffer. Bound protein was eluted with IMAC elution buffer and loaded onto the gel filtration column. Target protein containing fractions were pooled and TCEP was added to a final concentration of 2 mM. The protein was concentrated using an Amicon Ultra-15 centrifugal filter device with 10,000 NMWL (Millipore) to 16.9 mg/mL in a volume of 0.23 mL, and snap-frozen in liquid nitrogen.
**Crystallization**	The Protein solution was incubated with 10 mM ADP and 10 mM $MgCl_2$ for 1 hour. Crystals were obtained by the sitting drop vapor diffusion method. 0.1 µL protein solution was mixed with 0.1 µL of well solution consisting of 0.1 M HEPES pH 6.6, 0.2 M sodium thiocyanate, 20% w/v PEG 3350. The plate was incubated at 20 ºC and crystals appeared after 14 days. The crystals were transferred to a cryoprotectant solution consisting of 1 M HEPES pH 6.6, 0.2 M sodium thiocyanate, 22% w/v PEG 3350, 20% glycerol and flash frozen in liquid nitrogen.
**Data processing**	Intensity integration was performed by MOSFLM. SCALA was used for scaling. The structure was solved by molecular replacement using MOLREP with the structure of human DDX10 as search model (PDB: 2PL3). Structure refinement was performed with REFMAC 5.5.0102. The L168P mutation was detected by Mass spectrometry and DNA-sequencing. This part of the protein is not visible in the electron density

**Structure**	DDX20
**PDB Entry**	3B7G
**Entry clone accession**	gi|23270929
**Entry clone source**	Mammalian Gene Collection (BC034953)
**Tag**	N-terminal hexahistidine tag with integrated TEV protease cleavage site: mhhhhhhssgvdlgtenlyfq*sm
**Construct boundaries**	R41 – S248
**Construct sequence**	mhhhhhhssgvdlgtenlyfq*smRTAQDLSSPRTRTGDVLLAEPADFESLLLSRPVLEGLRAAGFERPSPVQLKAIPLG RCGLDLIVQAKSGTGKTCVFSTIALDSLVLENLSTQILILAPTREIAVQIHSVITAIGIKMEGLECHVFIGGTPLS QDKTRLKKCHIAVGSPGRIKQLIELDYLNPGSIRLFILDEADKLLEEGSFQEQINWIYSSLPASKQMLAVSATYP EFLANALTKYMRDPTFVRLNS
**Vector**	pNIC-Bsa4
**Expression host**	*E.coli* BL21-Gold(DE3)pRARE2, where BL21-Gold(DE3) cells (Stratagene) have been transformed with pRARE2 originating from the Rosetta2 strain (Novagen). The pRARE2 plasmid supplies tRNAs for rare codons.
**Growth method**	Cells from a glycerol stock were grown in 20 mL TB supplemented with 8 g/l glycerol, 100 µg/mL kanamycin and 34 µg/mL chloramphenicol at 30 ºC overnight. The overnight culture (20 mL) was used to inoculate 1.5 l TB supplemented with 8 g/l glycerol, 50 µg/mL kanamycin and approximately 200 µl BREOX FMT 30 anti-foam solution (Cognis Performance Chemicals UK Ltd). The culture was grown in a LEX bioreactor system (Harbinger Biotechnology) at 37 ºC until OD_{600} reached ~1.7. The culture was down-tempered to 18 ºC over a period of 1 hour before target expression was induced by addition of 0.5 mM IPTG. Expression was allowed to continue overnight and cells were harvested the following morning by centrifugation (5,500 x *g*, 10 min, 4 ºC). The resulting cell pellet (31.8 g wet cell weight) was resuspended in lysis buffer (1.5 mL/g cell pellet), supplemented with one tablet of Complete EDTA-free protease inhibitor (Roche Applied Science). The cell suspension was stored at -80 ºC.
**Extraction buffers**	Lysis buffer: 50 mM Na-phosphate, 500 mM NaCl, 10% glycerol, 10 mM imidazole, 0.5 mM TCEP, pH 7.5
**Extraction procedure**	The cell suspension was thawed and 2000 U Benzonase (Merck) were added. Cells were disrupted by sonication (Vibra-Cell, Sonics) at 80% amplitude for 3 min effective time (pulsed 4s on, 4s off) and cell debris was removed by centrifugation (49,100 x *g*, 20 min, 4 ºC). The supernatant was decanted and filtered through a 0.45 µm flask filter.
**Purification buffers**	IMAC wash1 buffer: 50 mM Na-phosphate, 500 mM NaCl, 10% glycerol, 10 mM imidazole, 0.5 mM TCEP, pH 7.5<br>IMAC wash2 buffer: 50 mM Na-phosphate, 500 mM NaCl, 10% glycerol, 25 mM imidazole, 0.5 mM TCEP, pH 7.5<br>IMAC elution buffer: 50 mM Na-phosphate, 500 mM NaCl, 10% glycerol, 500 mM imidazole, 0.5 mM TCEP, pH 7.5<br>Gel filtration (GF) buffer: 20 mM HEPES, 500 mM NaCl, 10% glycerol, 0.5 mM TCEP, pH 7.5
**Columns**	IMAC: Nickel-charged 1 mL HiTrap Chelating HP (GE Healthcare)<br>Gel filtration: HiLoad 16/60 Superdex 75 Prep Grade (GE Healthcare)
**Purification procedure**	Protein purification was performed as a two-step process on an ÄKTAxpress system (GE Healthcare) at 4°C. The IMAC column was charged with filtered lysate and washed with IMAC wash1 buffer followed by IMAC wash2 buffer. Bound protein was eluted with IMAC elution buffer and loaded onto the gel filtration column. Target protein containing fractions were pooled and TCEP was added to a final concentration of 2 mM. The protein was concentrated using an Amicon Ultra-15 centrifugal filter device with 10,000 NMWL (Millipore), and snap-frozen in liquid nitrogen.
**Post-purification**	**Tag removal**: The N-terminal histidine tag was proteolytically removed by incubating the protein with His-tagged TEV protease at a molar ratio of 50:1 at 4 ºC overnight. The proteolytic reaction did not reach completion, as judged by SDS-PAGE. Target protein was purified from uncleaved protein, tag and protease by passing the reaction mixture over an IMAC-column that was pre-equilibrated with the GF-Buffer containing 10 mM imidazole. The cleaved protein was concentrated and the buffer was exchanged to GF-Buffer, using a Vivaspin 20 centrifugal filter device with 10,000 MWCO (Sartorius). The final protein concentration was 33.8 mg/mL in a volume of 0.17 mL and the mass of the protein was confirmed by mass spectrometry.
**Crystallization**	Crystals were obtained by the sitting drop vapor diffusion method at 4 ºC. 0.1 µL protein solution (diluted to 20 mg/mL) including 20 mM ADP and 10 mM $MgCl_2$ was mixed with 0.1 µL of well solution consisting of 0.1 M Bis-Tris, pH 5.5, 0.2 M NaCl and 12% PEG 3350. Crystals appeared after one day and continued to grow for one more week. Crystals were briefly transferred to a cryoprotectant solution consisting of well solution complemented with 28% glycerol (replacing the same volume of water), and flash frozen in liquid nitrogen.
**Data processing**	Intensity integration was performed by XDS. Reflections were scaled with XSCALE. The structure was solved by molecular replacement using MOLREP and refined with REFMAC5.

**Structure**	DDX25
**PDB Entry**	2RB4
**Entry clone accession**	gil29792166
**Entry clone source**	Mammalian Gene Collection (BC050360)
**Tag**	N-terminal hexahistidine tag with integrated TEV protease cleavage site: mhhhhhhssgvdlgtenlyfq*sm
**Construct boundaries**	L307 – E479
**Construct sequence**	mhhhhhhssgvdlgtenlyfq*smLTLNNIRQYYVLCEHRKDKYQALCNIYGSITIGQAIIFCQTRRNAKWLTVEMIQDG HQVSLLSGELTVEQRASIIQRFRDGKEKVLITTNVCARGIDVKQVTIVVNFDLPVKQGEEPDYETYLHRIGRTG RFGKKGLAFNMIEVDELPSLMKIQDHFNSSIKQLNAEDMDEIE
**Vector**	pNIC-Bsa4
**Expression host**	*E.coli* BL21(DE3)
**Growth method**	Cells were grown in 50 mL TB supplemented with 8 g/L glycerol and 100 µg/mL kanamycin at 25 ºC overnight. The overnight culture was used to inoculate 4 * 0.75 L TB supplemented with 8 g/L glycerol, 50 µg/mL kanamycin. The flasks were incubated at 37°C on and shacked with 150 or 225 rpm. At an OD_{600} of 1.25 the cultures were down-tempered to 18 ºC over a period of 1 hour before target expression was induced by addition of 0.5 mM IPTG. Expression was allowed to continue overnight and cells were harvested the following morning by centrifugation (SLC-6000 rotor at 5000 rpm for 15 minutes, 4 ºC). The resulting cell pellet (60.3 g) was resuspended in 120 ml IMAC lysis buffer complemented with 2.5 tablets of Complete EDTA-free protease inhibitor (Roche Applied Science). The resuspended cells were frozen at -80°C.
**Extraction buffers**	IMAC lysis buffer: 50 mM Na Phosphate, 500 mM NaCl, 10% glycerol, pH 7.5, 0.5 mM TCEP, 10 mM imidazole
**Extraction procedure**	The cell suspension was thawed, 2000 U Benzonase (Merck) and 0.2 mM ADP was added. Cells were disrupted by sonication (Vibra-Cell, Sonics) at 30% amplitude for 3 min effective time (pulsed 20s on, 59s off) at 4°C. The cell debris was removed by centrifugation (SA-800 rotor, 20500 rpm, 20 min, 4 ºC). The supernatant was filtered through a 0.45 µm filter.
**Purification buffers**	IMAC wash 1 buffer: 50 mM Na Phosphate, 500 mM NaCl, 10% glycerol, pH 7.5, 0.5 mM TCEP, 10 mM imidazole<br>IMAC wash 2 buffer: 50 mM Na Phosphate, 500 mM NaCl, 10% glycerol, pH 7.5, 0.5 mM TCEP, 25 mM imidazole<br>IMAC elution buffer: 50 mM Na Phosphate, 500 mM NaCl, 10% glycerol, pH 7.5, 0.5 mM TCEP, 500 mM imidazole<br>Gel filtration buffer: 20 mM HEPES, 500 mM NaCl, 10% glycerol, pH 7.5, 2 mM TCEP
**Columns**	IMAC: Nickel-charged 1 mL HiTrap Chelating HP (GE Healthcare)<br>Gel filtration: HiLoad 16/60 Superdex 75 Prep Grade (GE Healthcare)
**Purification procedure**	Protein purification was performed as a two-step process on an ÄKTAxpress system (GE Healthcare) at 4°C. The IMAC column was charged with filtered lysate and washed with IMAC wash1 buffer followed by IMAC wash2 buffer. Bound protein was eluted with IMAC elution buffer and loaded onto the gel filtration column. Target protein containing fractions were pooled and TCEP was added to a final concentration of 2 mM. The protein was concentrated using an Amicon Ultra-15 centrifugal filter device with 10,000 NMWL (Millipore), and snap-frozen in liquid nitrogen.
**Post-purification**	**Tag removal**: The N-terminal histidine tag was proteolytically removed by incubating the protein with His-tagged TEV protease at a molar ratio of 15:1 at 4 ºC over two days. The proteolytic reaction reached completion, as judged by SDS-PAGE. Target protein was purified from uncleaved protein, tag and protease by passing the reaction mixture over an IMAC-column that was pre-equilibrated with the GF-Buffer containing 10 mM imidazole. 0.01 % of Detergent C12E8 was added and the cleaved protein was concentrated using an AmiconUltra device with MWCO of 10 000. The final protein concentration was 15.7 mg/mL in a volume of 0.22 mL. The mass of the protein was confirmed by mass spectrometry.
**Crystallization**	Crystals were obtained by the sitting drop vapor diffusion method at 20 ºC. Well solution consisted of 0.1 M Bis-Tris propane, 2.5 M Ammonium sulfate pH 6.5. Crystals appeared after one day, and were flash frozen in liquid nitrogen in well solution.
**Data processing**	Intensity integration was performed by XDS. Reflections were scaled with XSCALE. The structure was solved by molecular replacement using MOLREP (search model: pdb-entry 1FUK) and refined with REFMAC5.

**Structure**	DDX41
**PDB Entry**	2P6N
**Entry clone accession**	gi|21071032
**Entry clone source**	Mammalian Gene Collection (BC015476)
**Tag**	N-terminal hexahistidine tag with integrated TEV protease cleavage site: mhhhhhhssgvdlgtenlyfq*sm
**Construct boundaries**	G402 – G569
**Construct sequence**	mhhhhhhssgvdlgtenlyfq*smGAASLDVIQEVEYVKEEAKMVYLLECLQKTPPPVLIFAEKKADVDAIHEYLLLKG VEAVAIHGGKDQEERTKAIEAFREGKKDVLVATDVASKGLDFPAIQHV INYDMPEEIENYVHRIGRTGCSGNTGIATTFINKACDESVLMDLKALLLEAKQKVPPVLQVLHCG
**Vector**	pNIC-Bsa4
**Expression host**	*E.coli* BL21-Gold(DE3)pRARE2, where BL21-Gold(DE3) cells (Stratagene) have been transformed with pRARE2 originating from the Rosetta2 strain (Novagen). The pRARE2 plasmid supplies tRNAs for rare codons.
**Growth method**	Cells from a glycerol stock were grown in 20 mL TB supplemented with 8 g/l glycerol, 100 µg/mL kanamycin and 34 µg/mL chloramphenicol at 30 ºC overnight. The overnight culture (20 mL) was used to inoculate 1.5 l TB supplemented with 8 g/l glycerol, 50 µg/mL kanamycin and approximately 200 µl FMT 30 anti-foam (Cognis Performance Chemicals UK Ltd). The culture was grown in a LEX bioreactor system (Harbinger Biotechnology) at 37 ºC until OD_{600} reached ~2. The culture was down-tempered to 18 ºC over a period of 1 hour before target expression was induced by addition of 0.5 mM IPTG. Expression was allowed to continue overnight and cells were harvested the following morning by centrifugation (4,400 x *g*, 10 min, 4 ºC). The resulting cell pellet (14.4 g wet cell weight) was resuspended in lysis buffer (5 mL/g cell pellet), supplemented with one tablet of Complete EDTA-free protease inhibitor (Roche Applied Science). The cell suspension was stored at -80 ºC.
**Extraction buffers**	Lysis buffer: 100 mM HEPES, 500 mM NaCl, 10% glycerol, 10 mM imidazole, 0.5 mM TCEP, pH 8.0.
**Extraction procedure**	The cell suspension was thawed and 2000 U Benzonase (Merck) was added. Cells were disrupted by sonication (Vibra-Cell, Sonics) at 80% amplitude for 3 min effective time (pulsed 4s on, 4s off) and cell debris was removed by centrifugation (49,000 x *g*, 20 min, 4 ºC). The supernatant was filtered through a 0.45 µm filter.
**Purification buffers**	IMAC wash1 buffer: 20 mM HEPES, 500 mM NaCl, 10% glycerol, 10 mM imidazole, 0.5 mM TCEP, pH 7.5<br>IMAC wash2 buffer: 20 mM HEPES, 500 mM NaCl, 10% glycerol, 25 mM imidazole, 0.5 mM TCEP, pH 7.5<br>IMAC elution buffer: 20 mM HEPES, 500 mM NaCl, 10% glycerol, 500 mM imidazole, 0.5 mM TCEP, pH 7.5<br>Gel filtration (GF) buffer: 20 mM HEPES, 500 mM NaCl, 10% glycerol, 0.5 mM TCEP, pH 7.5
**Columns**	IMAC: Nickel-charged 1 mL HiTrap Chelating HP (GE Healthcare)<br>Gel filtration: HiLoad 16/60 Superdex 75 Prep Grade (GE Healthcare)
**Purification procedure**	Protein purification was performed as a two-step process on an ÄKTAxpress system (GE Healthcare) at 4°C. The IMAC column was charged with filtered lysate and washed with IMAC wash1 buffer followed by IMAC wash2 buffer. Bound protein was eluted with IMAC elution buffer and loaded onto the gel filtration column. Target protein containing fractions were pooled and TCEP was added to a final concentration of 2 mM. The protein was concentrated using an Amicon Ultra-15 centrifugal filter device with 10,000 NMWL (Millipore) to 19.1 mg/mL in a volume of 0.25 mL, and snap-frozen in liquid nitrogen.
**Crystallization**	Crystals were obtained by the sitting drop vapor diffusion method. 0.1 µL protein solution (19.1 mg/mL) was mixed with 0.2 µL of well solution consisting of 0.1 M bis-Tris pH 5.5, 0.2 M lithium sulfate and 25% (w/v) PEG 3350. The plate was incubated at 20 ºC. Crystals appeared within three days and continued to grow for ten more days to reach their maximal size (approx. 120 µm × 50 µm × 50 µm). The crystals were transferred to a cryoprotectant solution consisting of well solution with 25% glycerol (replacing the same volume of water), and flash frozen in liquid nitrogen.
**Data processing**	Intensity integration was performed by XDS. Reflections were scaled with XSCALE. The structure was solved by molecular replacement using the helicase domain from the human protein DDX3X as a search model (PDB entry: 2I4I) with the program MOLREP. Pseudo translation symmetry was detected. REFMAC5 was used for refinement and Coot for model building. NCS and TLS restrained refinement was used. The TLS groups were selected using the tlsmd server http://skuld.bmsc.washington.edu/~tlsmd/.

**Structure**	DDX47
**PDB Entry**	3BER
**Entry clone accession**	gi|45786091
**Entry clone source**	Mammalian Gene Collection (BC068009)
**Tag**	N-terminal hexahistidine tag with integrated TEV protease cleavage site: mhhhhhhssgvdlgtenlyfq*sm
**Construct boundaries**	E5 – S230
**Construct sequence**	mhhhhhhssgvdlgtenlyfq*smEEHDSPTEASQPIVEEEETKTFKDLGVTDVLCEACDQLGWTKPTKIQIEAIPLALQ GRDIIGLAETGSGKTGAFALPILNALLETPQRLFALVLTPTRELAFQISEQFEALGSSIGVQSAVIVGGIDSMSQS LALAKKPHIIIATPGRLIDHLENTKGFNLRALKYLVMDEADRILNMDFETEVDKILKVIPRDRKTFLFSATMTK KVQKLQRAALKNPVKCAVSS
**Vector**	pNIC-Bsa4
**Expression host**	*E.coli* BL21-Gold(DE3)pRARE2, where BL21-Gold(DE3) cells (Stratagene) have been transformed with pRARE2 originating from the Rosetta2 strain (Novagen). The pRARE2 plasmid supplies tRNAs for rare codons.
**Growth method**	Cells were grown in 20 mL TB supplemented with 8 g/l glycerol, 100 µg/mL kanamycin and 34 µg/mL chloramphenicol were grown at 30 ºC overnight. The overnight culture was used to inoculate 1.5 l TB supplemented with 8 g/l glycerol, 50 µg/mL kanamycin, 34 µg/mL chloramphenicol and approximately 250 µl PPG P2000 anti-foam solution (Fluka). The culture was grown in a LEX bioreactor system (Harbinger Biotechnology) at 37 ºC until OD_{600} reached ~1.2. The culture was down-tempered to 18 ºC over a period of 1 hour before target expression was induced by addition of 0.5 mM IPTG. Expression was allowed to continue overnight and cells were harvested the following morning by centrifugation (5,500 x *g*, 15 min, 4 ºC). The resulting cell pellet (18.9 g wet cell weight) was resuspended in lysis buffer (1 mL/g cell pellet), supplemented with 0.5 tablet of Complete EDTA-free protease inhibitor (Roche Applied Science). The cell suspension was stored at -80 ºC.
**Extraction buffers**	Lysis buffer: 50 mM HEPES, 500 mM NaCl, 10% glycerol, 10 mM imidazole, 0.5 mM TCEP, pH 7.8
**Extraction procedure**	The cell suspension was thawed and 1000 U Benzonase (Merck), 2 mM ADP and 2 mM $MgCl_2$ were added to the cell suspension. The cells were disrupted by sonication (Vibra-Cell, Sonics) at 80% amplitude for 3 min effective time (pulsed 4s on, 4s off). Cell debris was removed by centrifugation (49,100 x *g*, 20 min, 4 ºC) and the supernatant was filtered through a 0.45 µm filter.
**Purification buffers**	IMAC wash1 buffer: 30 mM HEPES, 500 mM NaCl, 10% glycerol, 10 mM imidazole, 0.5 mM TCEP, pH 7.5<br>IMAC wash2 buffer: 30 mM HEPES, 500 mM NaCl, 10% glycerol, 25 mM imidazole, 0.5 mM TCEP, pH 7.5<br>IMAC elution buffer: 30 mM HEPES, 500 mM NaCl, 10% glycerol, 500 mM imidazole, 0.5 mM TCEP, pH 7.5<br>Gel filtration (GF) buffer: 30 mM HEPES, 500 mM NaCl, 10% glycerol, 0.5 mM TCEP, pH 7.5
**Columns**	IMAC: Nickel-charged 1 mL HiTrap Chelating HP (GE Healthcare)<br>Gel filtration: HiLoad 16/60 Superdex 75 Prep Grade (GE Healthcare)
**Purification procedure**	Protein purification was performed as a two-step process on an ÄKTAxpress system (GE Healthcare) at 4°C. The IMAC column was charged with filtered lysate and washed with IMAC wash1 buffer followed by IMAC wash2 buffer. Bound protein was eluted with IMAC elution buffer and loaded onto the gel filtration column. Target protein containing fractions were pooled and TCEP was added to a final concentration of 2 mM. The protein was concentrated using an Amicon Ultra-15 centrifugal filter device with 10,000 NMWL (Millipore) to 17.6 mg/mL in a volume of 0.13 mL, and snap-frozen in liquid nitrogen.
**Crystallization**	Crystals were obtained by the sitting drop vapor diffusion method. 0.1 µL protein solution (diluted to 17 mg/mL) including 5 mM ADP and 5 mM $MgCl_2$ was mixed with 0.2 µL of well solution consisting of 0.1 M Na/K-phosphate pH 5.2, 0.05 M NaCl and 42.5% (v/v) PEG 200. The plate was incubated at 4 ºC. A crystal appeared after three weeks and continued to grow for two more weeks to reach maximal size (approx. 200 µm × 140 µm × 30 µm). The crystal was picked directly from the drop and flash frozen in liquid nitrogen without cryoprotectant.
**Data processing**	Intensity integration was performed by XDS. Reflections were scaled with XSCALE. The structure was solved by molecular replacement using MOLREP and the PDB entry 2GXS as a search model. Automated model building was performed using ARP/wARP. Refmac5 was used for refinement and Coot for model building.

**Structure**	DDX52
**PDB Entry**	3DKP
**Entry clone accession**	gi|27697141
**Entry clone source**	Mammalian Gene Collection (BC041785)
**Tag**	N-terminal hexahistidine tag with integrated TEV protease cleavage site: mhhhhhhssgvdlgtenlyfq*sm
**Construct boundaries**	K139 – S381
**Construct sequence**	mhhhhhhssgvdlgtenlyfq*smKINFLRNKHKIHVQGTDLPDPIATFQQLDQEYKINSRLLQNILDAGFQMPTPIQMQAIPVMLHGRELLASAPTGSGKTLAFSIPILMQLKQPANKGFRALIISPTRELASQIHRELIKISEGTGFRIHMIHKAAVAAKKFGPKSSKKFDILVTTPNRLIYLLKQDPPGIDLASVEWLVVDESDKLFEDGKTGFRDQLASIFLACTSHKVRRAMFSATFAYDVEQWCKLNLDNVISVSIGARNS
**Vector**	pNIC-Bsa4
**Expression host**	*E.coli* BL21(DE3) R3 pRARE, where R3 denotes a derivative of BL21(DE3) resistant to a strain of T1 bacteriophage (SGC Oxford) and the pRARE plasmid originating from the Rosetta strain (Novagen) supplies tRNAs for rare codons.
**Growth method**	Cells from a glycerol stock were grown in 30 mL TB supplemented with 8 g/l glycerol, 100 µg/mL kanamycin and 34 µg/mL chloramphenicol at 30 ºC overnight. The overnight culture (20 mL) was used to inoculate 1.5 l TB supplemented with 8 g/l glycerol, 50 µg/mL kanamycin and approximately 200 µl PPG P2,000 81380 anti-foam solution (Fluka). The culture was grown in a LEX bioreactor system (Harbinger Biotechnology) at 37 ºC until OD_{600} reached ~2. The bottle was down-tempered to 18 ºC over a period of 1 hour before target expression was induced by addition of 0.5 mM IPTG. Expression was allowed to continue overnight and cells were harvested the following morning by centrifugation (4,400 x *g*, 10 min, 4 ºC). The resulting cell pellet (33.2 g wet cell weight) was resuspended in lysis buffer (1.5 mL/g cell pellet), supplemented with 2500 U Benzonase (Merck) and 1 tablet of Complete EDTA-free protease inhibitor (Roche Applied Science). The cell suspension was stored at -80 ºC.
**Extraction buffers**	Lysis buffer: 100 mM HEPES, 500 mM NaCl, 10% glycerol, 10 mM imidazole, 0.5 mM TCEP, pH 8.0
**Extraction procedure**	The cell suspension was thawed and diluted to 2 x 68 mL with lysis buffer. Cells were disrupted by sonication (Vibra-Cell, Sonics) at 80% amplitude for 3 min effective time (pulsed 4s on, 4s off) and cell debris was removed by centrifugation (49,000 x *g*, 20 min, 4 ºC). The supernatant was filtered through a 0.45 µm filter.
**Purification buffers**	IMAC wash1 buffer: 20 mM HEPES, 500 mM NaCl, 10% glycerol, 10 mM imidazole, 0.5 mM TCEP, pH 7.5<br>IMAC wash2 buffer: 20 mM HEPES, 500 mM NaCl, 10% glycerol, 25 mM imidazole, 0.5 mM TCEP, pH 7.5<br>IMAC elution buffer: 20 mM HEPES, 500 mM NaCl, 10% glycerol, 500 mM imidazole, 0.5 mM TCEP, pH 7.5<br>Gel filtration (GF) buffer: 20 mM HEPES, 500 mM NaCl, 10% glycerol, 0.5 mM TCEP, pH 7.5
**Columns**	IMAC: Nickel-charged 1 mL HiTrap Chelating HP (GE Healthcare)<br>Gel filtration: HiLoad 16/60 Superdex 200 Prep Grade (GE Healthcare)
**Purification procedure**	Protein purification was performed as a two-step process on an ÄKTAxpress system (GE Healthcare) at 4°C. The IMAC column was charged with filtered lysate and washed with IMAC wash1 buffer followed by IMAC wash2 buffer. Bound protein was eluted with IMAC elution buffer and loaded onto the gel filtration column. Target protein containing fractions were pooled and TCEP was added to a final concentration of 2 mM. The protein was concentrated using an Amicon Ultra-15 centrifugal filter device with 10,000 NMWL (Millipore) to 49.9 mg/mL in a volume of 1.5 mL. ADP and $MgCl_2$ were added to final concentrations of 5 mM and 10 mM respectively. Protein was snap-frozen in liquid nitrogen.
**Post-purification**	**Tag removal**: The N-terminal histidine tag was proteolytically removed by incubating the target protein with His-tagged TEV protease at a molar ratio of 30:1 at 4 ºC overnight. The proteolytic reaction went to completion, as judged by SDS-PAGE. Target protein was purified from tag and protease by passing the reaction mixture over an IMAC column pre-equilibrated with IMAC wash1 buffer. The cleaved protein was concentrated and the buffer was changed to GF buffer using an Amicon Ultra-15 centrifugal filter device with 5,000 NMWL (Millipore). The final protein concentration was determined to 20.7 mg/mL in a volume of 0.46 mL. The mass of the protein was confirmed by mass spectrometry.
**Crystallization**	Crystals were obtained by the sitting drop vapor diffusion method. 0.1 µL of the protein solution (diluted to 18.4 mg/mL) including 20 mM ADP and 10 mM $MgCl_2$ was mixed with 0.2 µL of well solution consisting of 0.1 M imidazole, pH 8.0 and 10% (w/v) PEG 8000. The plate was incubated at 4 ºC and crystals appeared after two days. The crystal was quickly transferred to cryoprotectant solution consisting of 0.1 M imidazole, pH 8.0, 12% (w/v) PEG 800 and 30% glycerol, and flash-frozen in liquid nitrogen.
**Data processing**	Intensity integration was performed by XDS. Reflections were scaled with XSCALE. The structure was solved by molecular replacement using MOLREP with the pdb-entry 2DB3 as a search model. The structure was refined with REFMAC5 using 3 TLS groups for the protein chain.

**Structure**	DDX53
**PDB Entry**	3IUY
**Entry clone accession**	gi|45709415
**Entry clone source**	Mammalian Gene Collection (BC067878)
**SGC clone accession**	DDX53A-s001
**Tag**	N-terminal hexahistidine tag with integrated TEV protease cleavage site: mhhhhhhssgvdlgtenlyfq*sm
**Construct boundaries**	T204 – V430
**Construct sequence**	mhhhhhhssgvdlgtenlyfq*smTCDDLKSGEKRLIPKPTCRFKDAFQQYPDLLKSIIRVGILKPTPIQSQAWPIILQGID LIVVAQTGTGKTLSYLMPGFIHLDSQPISREQRNGPGMLVLTPTRELALHVEAECSKYSYKGLKSICIYGGRN RNGQIEDISKGVDIIIATPGRLNDLQMNNSVNLRSITYLVIDEADKMLDMEFEPQIRKILLDVRPDRQTVMTSA TWPDTVRQLALSYLKDPMIVYV
**Vector**	pNIC-Bsa4
**Expression host**	*E. coli* BL21(DE3) R3 pRARE, where R3 denotes a derivative of BL21(DE3) resistant to a strain of T1 bacteriophage (SGC Oxford) and the pRARE plasmid originating from the Rosetta strain (Novagen) supplies tRNAs for rare codons.
**Growth method**	Cells from a glycerol stock were grown in 50 mL TB supplemented with 8 g/l glycerol, 100 µg/mL kanamycin and 34 µg/mL chloramphenicol at 37 ºC overnight. The overnight culture (50 mL) was used to inoculate 4.5 l TB (divided into 3 x 1.5 l bottles) supplemented with 8 g/l glycerol, 50 µg/mL kanamycin and approximately 200 µl 204 Antifoam A6426 (Sigma-Aldrich) per bottle. The culture was grown in a LEX bioreactor system (Harbinger Biotechnology) at 37 ºC until OD_{600}nm had reached 1.6. The culture was down-tempered to 18 ºC over a period of 1 hour before target expression was induced by addition of 0.5 mM IPTG. Expression was allowed to continue overnight and cells were harvested the following morning by centrifugation (4,400 x g, 10 min, 4 ºC). The resulting cell pellet (110 g wet cell weight) was stored at -80 ºC.
**Extraction buffers**	100 mM HEPES, 500 mM NaCl, 10% glycerol, 10 mM imidazole, 0.5 mM TCEP, pH 8.0
**Extraction procedure**	The resulting cell pellet was resuspended in lysis buffer (1.5 mL/g cell pellet), supplemented with Protease-Inhibitors[1] and Benzonase[1] at 4°C. The resuspended cells were stored in falcon tubes in the -80 freezer. Cells were disrupted by sonication (Vibra-Cell, Sonics) at 80% amplitude for 3 min effective time (pulsed 4s on, 4s off) and cell debris was removed by centrifugation (49,000 x g, 40 min, 4 ºC). The supernatant was decanted and filtered through a 0.45 µm flask filter.<br>[1]Complete stock solution: 1 tablet Complete EDTA-free (protease inhibitor) and 8 µL Benzonase (2000 U) dissolved in 1 mL buffer. 1 mL complete stock solution is added to cells from 1.5 L cell culture.
**Purification buffers**	IMAC lysis buffer: 100 mM HEPES, 500 mM NaCl, 10% glycerol, 10 mM imidazole, 0.5 mM TCEP, pH 8.0<br>IMAC wash 1 buffer: 20 mM HEPES, 500 mM NaCl, 10% glycerol, 10 mM imidazole, 0.5 mM TCEP, pH 7.5<br>IMAC wash 2 buffer: 20 mM HEPES, 500 mM NaCl, 10% glycerol, 25 mM imidazole, 0.5 mM TCEP, pH 7.5<br>IMAC elution buffer: 20 mM HEPES, 500 mM NaCl, 10% glycerol, 500 mM imidazole, 0.5 mM TCEP, pH 7.5<br>Gel filtration buffer: 20 mM HEPES, 300 mM NaCl, 10% glycerol, 0.5 mM TCEP, pH 7.5
**Columns**	IMAC: Nickel-charged 5 mL HisTrap HP (GE Healthcare); Gel filtration column: HiLoad 16/60 Superdex 75 Prep Grade (GE Healthcare)
**Purification procedure**	Protein purification was performed as a two-step process on an ÄKTAxpress system (GE Healthcare) at 4°C. The IMAC column was charged with filtered lysate and washed with IMAC wash1 buffer followed by IMAC wash2 buffer. Bound protein was eluted with IMAC elution buffer and loaded onto the gel filtration column. Target protein containing fractions were pooled and TCEP was added to a final concentration of 2 mM. The protein was concentrated using an Amicon Ultra-15 centrifugal filter device with 10,000 NMWL (Millipore), and snap-frozen in liquid nitrogen.
**Post-purification**	**Tag removal**: The Protein sample was incubated with His-tagged TEV protease in a molar ratio of 30:1 at 4 ºC for 2 days. The proteolytic reaction went to completion, as judged by SDS-PAGE. The DDX53 was purified from tag and protease by passing the reaction mixture over an IMAC column pre-equilibrated with IMAC wash1 buffer. The protein fraction was concentrated in a Vivaspin concentrator (Sartorius) with 10 000 MWCO. The buffer was exchanged to GF-buffer in this concentrator.
**Crystallization**	Crystals were obtained by the sitting drop vapor diffusion method at 4 ºC. 0.1 µL protein solution (pre-incubated with 20 mM ADP and 1 mM $MgCl_2$ for 1 hour) was mixed with 0.2 µL of well solution consisting of 0.1M MES, 25% PEG 6000, 0.2 M ammonium chloride. Crystals appeared between 3 and 5 days. The crystals were transferred to a cryoprotectant solution consisting of 27% PEG6000, 0.1M MES pH 6, 0.2M ammonium chloride, 15% glycerol, 0.2M NaCl, and flash frozen in liquid nitrogen.
**Data processing**	Intensity integration was performed by MOSFLM. Two datasets were combined and then scaled with SCALA. The structure was solved by molecular replacement using MOLREP with the structure of human DDX5 as template (PDB: 3FE2). Structure refinement was performed with REFMAC 5.5.0035.
